# Photo-Induced, Phenylhydrazine-Promoted Transition-Metal-Free Dehalogenation of Aryl Fluorides, Chlorides, Bromides, and Iodides

**DOI:** 10.3390/molecules28196915

**Published:** 2023-10-03

**Authors:** Yiwei Zhu, Zhimin Wu, Hongcai Sun, Junjun Ding

**Affiliations:** School of Chemistry and Environmental Engineering, Anhui Polytechnic University, Wuhu 241000, Chinadingjunjun@nexchip.com.cn (J.D.)

**Keywords:** photo-induced hydrogenation, radical-mediated hydrogenation, aryl halides

## Abstract

In this study, we present a straightforward and highly effective photo-triggered hydrogenation method for aryl halides, devoid of transition-metal catalysts. Through the synergistic utilization of light, PhNHNH_2_, and a base, we have successfully initiated the desired radical-mediated hydrogenation process. Remarkably, utilizing mild reaction conditions, a wide range of aryl halides, including fluorides, chlorides, bromides, and iodides, can be selectively transformed into their corresponding (hetero)arene counterparts, with exceptional yields. Additionally, this approach demonstrates a remarkable compatibility with diverse functional groups and heterocyclic compounds, highlighting its versatility and potential for use in various chemical transformations.

## 1. Introduction

Reductive hydrodehalogenation, an important chemical transformation, finds extensive applications in environmental protection, medicinal chemistry, and organic synthesis. This process plays a critical role in modifying bioactive molecules with halogen groups during the late stages, enabling precise structural adjustment. Moreover, it assumes a vital role in converting hazardous and long-lasting halogenated organic pollutants into eco-friendly compounds [1,2,3,4,5,6,7,8,9].

To address the significance of hydrodehalogenation, organic chemists have dedicated considerable efforts over the decades to developing efficient and straightforward methods. Traditional methods typically employ AIBN as an initiator for the radical reductive dehalogenation of aryl halides. Additionally, transition-metal-catalyzed reductions using diverse agents, such as H_2_, hydrosilanes, hydrides, and alcohols, are extensively applied in this context. Furthermore, scholars have explored the dehalogenation of aryl or alkyl halides through electrolysis, wherein a metal electrode is employed to provide electrons. They have also studied photo-induced dehalogenation, which involves using amines as terminal reductants and hydrogen atom donors, in combination with organic or inorganic photocatalysts and various additives [10,11,12,13,14,15,16,17,18,19,20,21,22].

However, these approaches are accompanied by inherent constraints, such as an inadequate selectivity resulting in diminished yields, rigorous reaction conditions, dependency on costly noble metals, the necessity of inert atmospheres and specific ligands, an excessive utilization of potentially hazardous radical initiators, and the requirement for specialized photo/electrochemical reactors. Therefore, there is significant interest in developing a straightforward and highly efficient dehalogenation method that utilizes light as a trigger, aiming to avoid the use of expensive metal catalysts, complex additives, and specialized experimental setups [23,24,25,26,27,28,29,30,31,32,33,34,35,36,37,38].

## 2. Results and Discussion

An exploration of reaction conditions was conducted, employing the dehalogenation of 4-chloro-1,1′-biphenyl 1a as a representative model reaction. A comprehensive summary of the findings is presented in Table 1. The initial attempts, which used four equivalents of *t-*BuOK and eight equivalents of Et_3_N, while exposing the mixture to blue LED light (365 nm, 28 W), yielded only 6% of the dechlorinated product 2a (entry 1). However, the introduction of PhNHNH_2_ into the system significantly improved the dichlorination outcome (entries 2–5). To explore the impact of different solvents on the dichlorination process, various solvents, including MeCN, 1,4-Dioxane, and *t*-BuOH, were evaluated (entries 6–8), resulting in dichlorination yields ranging from 15% to 38%. Different light sources, such as 254 nm, 395 nm, 405 nm, 455 nm, and 485 nm, and even the absence of light (dark condition), were also examined, but none surpassed the efficiency of 365 nm blue LED irradiation (entries 9–14). Furthermore, investigations into the reaction without *t*-BuOK or Et_3_N present were conducted, which led to 8% and 26% yields of 2a, respectively. To optimize the reaction conditions further, different base combinations were tested (entries 15–19), among which 8 eq. of *t*-BuOK in combination with 16 eq. of *N,N*′-Dimethyl-1,2-ethanediamine proved to be the most efficient. The effects of lowering the reaction temperature were also evaluated (entry 20), but this resulted in a decrease in the dichlorination yield. After a comprehensive comparison of all the conditions in Table 1, the reaction conditions presented in entry 19 were carefully chosen as the most optimal, due to their outstanding performance.

**Scheme 1:**
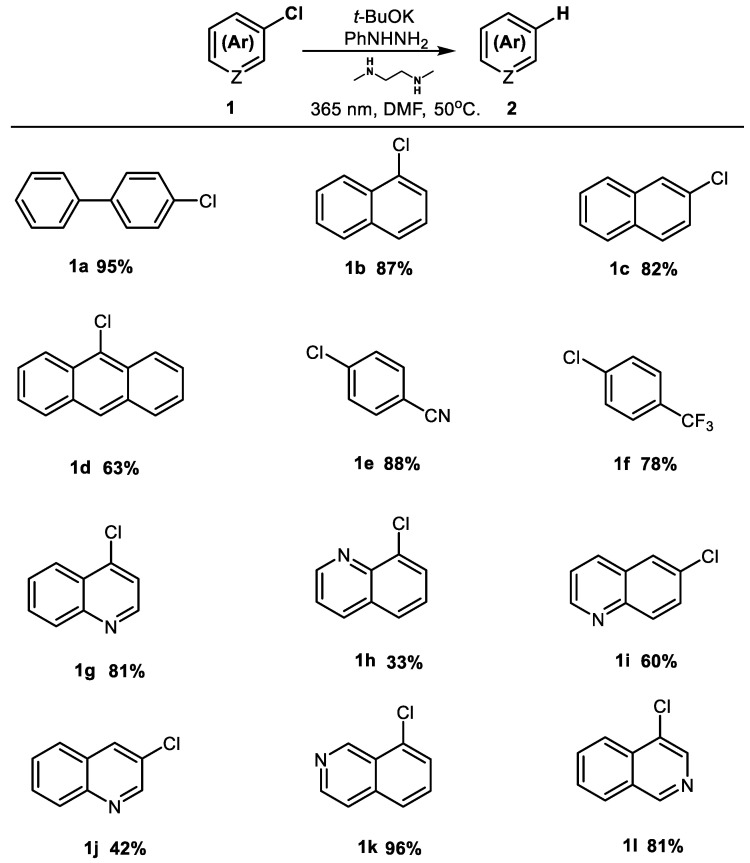
The dehalogenation of aryl chlorides ^a^. ^a^ Conditions: 1 (0.2 mmol), PhNHNH_2_ (0.8 mmol), *t-*BuOK (1.6 mmol), *N,N*′*-Dimethyl-1,2-ethanediamine* (3.2 mmol), dry DMF (3 mL), 50 °C, blue LED (365 nm, 28 W), sealed tube, 48 h, isolated yields.

Having established the optimized conditions, we extensively investigated the reactivity of diverse aryl chlorides (Scheme 1). Under standard conditions, smooth reactions were notably observed for biphenyl (1a), naphthyl (1b, c), and anthracenyl (1d) chlorides, resulting in impressive yields ranging from 63% to 95% for the desired reduction products. Additionally, aryl halides bearing cyano and trifluoromethyl substituents (1e, f) displayed a favorable reactivity, delivering the corresponding arenes in yields of 88% and 78%, respectively. Furthermore, the reaction exhibited a remarkable tolerance toward chlorinated heteroarenes, including chloroquinoline (1g–j) and chloroisoquinoline (1k, l).

**Scheme 2:**
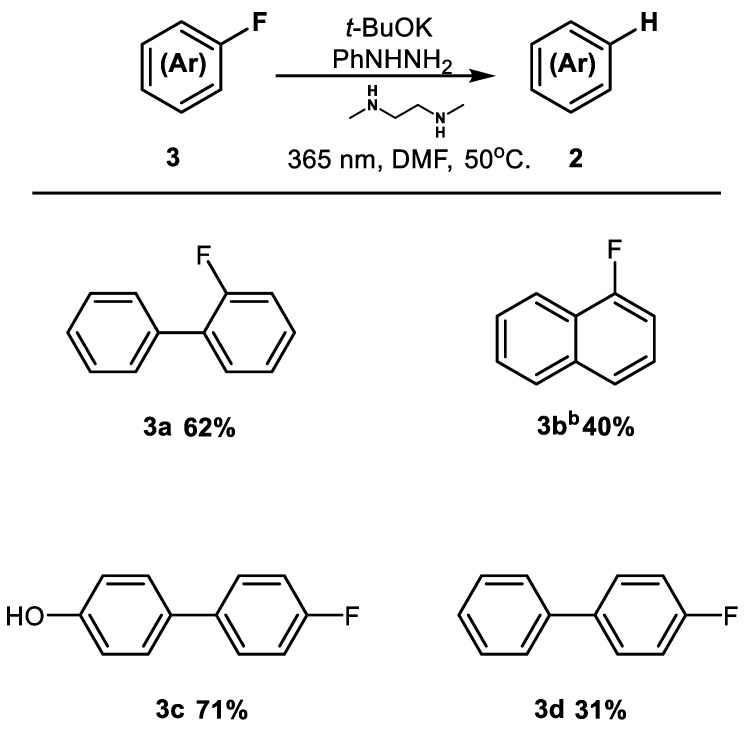
The dehalogenation of aryl fluorides ^a^. ^a^ Conditions: 3 (0.2 mmol), PhNHNH_2_ (0.8 mmol), *t-*BuOK (1.6 mmol), *N,N′-Dimethyl-1,2-ethanediamine* (3.2 mmol), dry DMF (3 mL), 50 °C, blue LED (365 nm, 28 W), sealed tube, 96 h, isolated yields. ^b^ A mixture of DMSO (1.5 mL) and DMF (1.5 mL) was used as a solvent.

The hydrodefluorination of organic fluorides represents a valuable strategy, not only for detoxifying environmentally hazardous halogenated chemicals but also for its significant potential in molecular functionalization, especially in the field of drug discovery. Despite the rapid progress in hydrodefluorination methodologies, the light-promoted hydrodefluorination of aryl fluorides remains relatively scarce. However, upon subjecting aryl fluorides 3a–d to the specified standard conditions outlined in Table 1 (entry 19), we observed a smooth hydrodefluorination process, leading to the formation of the corresponding arenes in favorable yields (Scheme 2). This encouraging outcome indicates the promising utility of this approach in both organic synthesis and pharmaceutical applications.

**Scheme 3:**
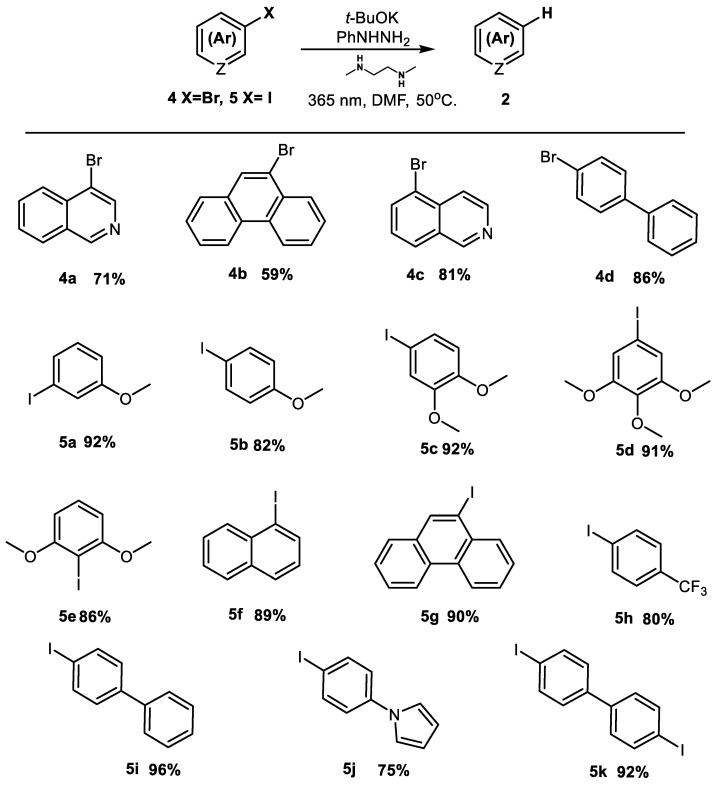
The dehalogenation of aryl bromides and iodides ^a^. ^a^ Conditions: 4 or 5 (0.2 mmol), PhNHNH_2_ (0.8 mmol), *t-*BuOK (1.6 mmol), *N,N’-Dimethyl-1,2-ethanediamine* (3.2 mmol), dry DMF (3 mL), 50 °C, blue LED (365 nm, 28 W), sealed tube, 48 h, isolated yields.

In order to comprehensively explore the applicability of this system, we conducted additional investigations involving aryl bromide 4 and aryl iodide 5. The outcomes of these experiments are succinctly presented in Scheme 3. Aryl bromides 4a–d and aryl iodides 5a–k all underwent efficient reduction, with yields ranging from good to excellent, which is encouraging. These results provide additional compelling evidence supporting the efficiency and versatility of this transition-metal-free approach to dehalogenating diverse aryl halides under mild reaction conditions.

**Scheme 4:**
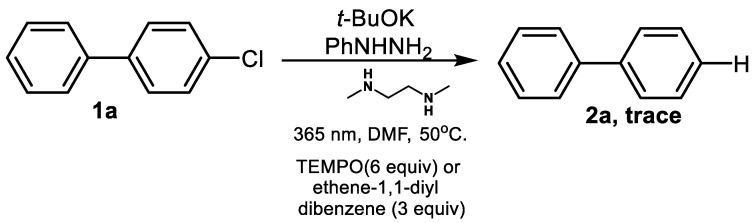
Free radical trap and control experiments.

To elucidate the underlying mechanism governing this dehalogenation reaction, a series of insightful control experiments was meticulously conducted. The addition of 1 equivalent of the standard radical scavenger TEMPO led to a reduction in the yield of 2a under the specified conditions.Furthermore, a progressive decrease in the yield of 2a was observed with an increased amount of TEMPO, eventually leading to the complete inhibition of the reaction upon the introduction of TEMPO (Scheme 4). Moreover, subjecting 1,1-diphenylethylene to the standard reaction conditions led to the complete inhibition of the model reaction in the radical trapping experiment. The radical trapping experiments provide compelling evidence supporting the theory that the dehalogenation reaction can be explained by a credible radical mechanism.

**Scheme 5:**
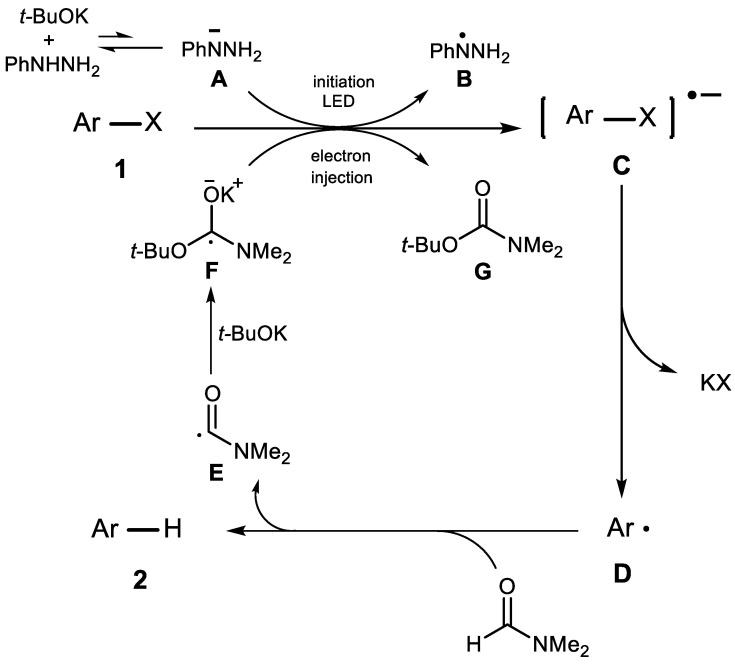
The plausible mechanism.

Based on the insights gleaned from the radical trapping experiments, we propose a plausible mechanism, which is illustrated and substantiated in Scheme 5. The reaction initiates with the deprotonated form of PhNHNH_2_ (A), which donates an electron to aryl halide 1 under the influence of LED light. This electron transfer results in the formation of radical anion C and radical B. Subsequently, aryl radical D captures a hydrogen atom from DMF, yielding product 2 and releasing E. E undergoes attack from *t*-BuOK, forming radical anion F. It is worth noting that radical anion F plays a crucial role as the principal electron donor in the subsequent stages of the reaction [23,24,25,26,27,28,29,30,31,32].

## 3. Conclusions

In this study, we introduce a novel and straightforward strategy for the transition-metal-free, light-driven hydrogenation of aryl halides. The key to initiating this radical-mediated hydrogenation lies in the synergistic use of PhNHNH_2_, LED light, and a suitable base system. Notably, a wide range of aryl halides efficiently underwent reduction, yielding the corresponding products in yields ranging from moderate to excellent, all under mild reaction conditions. The method exhibits a remarkable tolerance toward various functional groups and heterocyclic compounds, underscoring its practicality in synthetic applications.

## 4. Materials and Methods

### 4.1. General Information

All reagents and solvents utilized in this study were procured from commercial sources and employed without further purification, unless otherwise specified. Nuclear magnetic resonance (NMR) spectra were acquired using Bruker AV300, Bruker AV400, and Bruker AV500M spectrometers, and chemical shifts were reported in parts per million (δ) relative to the internal standard, tetramethylsilane (TMS), positioned at 0 ppm in CDCl_3_. The determinations of some chemicals were compared with the spectra reported in the literature. The light sources employed were LED lamps with wavelengths ranging from 254 nm to 485 nm, providing an output power of 7 W. The reaction progress was monitored via thin-layer chromatography (TLC). Column chromatography was performed using silica gel (200–300 mesh), and the compounds were visualized under ultraviolet light. Elution during column chromatography was achieved using a mixture of ethyl acetate and petroleum ether as the eluent. High-performance liquid chromatography (HPLC) analyses were conducted using a Shimadzu LC-16 spectrometer. Gas-chromatography–mass-spectrometry (GC-MS) analyses were carried out utilizing a Thermo TRACE 1300 ISQ LT spectrometer.

### 4.2. Procedure for the Deiodination of 4-chloro-1,1′-biphenyl

A 35 mL thick-walled pressure vessel equipped with a Teflon cap was charged with a magnetic stirring bar, 4-chloro-1,1’-biphenyl (0.2 mmol), *t-*BuOK (1.6 mmol), PhNHNH_2_ (0.8 mmol), *N,N’-*Dimethyl-1,2-ethanediamine (3.2 mmol), and dry DMF (3 mL). Subsequently, the pressure vessel was immersed in an oil bath along with LED lamps (365 nm, 7 W × 4) at a temperature of 50 °C. To ensure light exclusion, the entire reaction system was carefully shielded with tinfoil film. The reaction mixture was stirred for 48 h. Following the designated reaction time, 0.2 mmol of 4-methylbiphenyl was introduced into the pressure vessel. Subsequently, 10 mL of water was added to terminate the reaction, and the resulting mixture was extracted using ethyl acetate (5 mL × 4). The combined organic extracts were washed with brine, dried with sodium sulfate, and then subjected to filtration for the subsequent HPLC analysis.

### 4.3. General Procedure for the Reduction of C−X Bond

A 35 mL thick-walled pressure vessel, equipped with a Teflon cap and a magnetic stirring bar, was charged with aryl halide (0.2 mmol), *t-*BuOK (1.6 mmol), PhNHNH_2_ (0.8 mmol), *N,N’-*Dimethyl-1,2-ethanediamine (3.2 mmol), and dry DMF (3 mL). The pressure vessel was then positioned in an oil bath, accompanied by LED lamps emitting light at a wavelength of 365 nm and a power output of 7 W × 2, while maintaining a temperature of 50 °C. To ensure light exclusion, the entire reaction system was meticulously wrapped with tinfoil film. The mixture was stirred for 48 or 96 h, as specified. Following the designated reaction time, 10 mL of water was added to quench the reaction, and the resulting mixture was extracted using ethyl acetate (5 mL × 4). The combined organic extracts were subsequently washed with brine, dried with sodium sulfate, and concentrated under reduced pressure. The resulting residue was purified via preparative TLC on silica gel, using a gradient elution of petroleum ether and ethyl acetate (300:1–5:1) as the eluent, to yield the desired products. The confirmation of the products was accomplished via comparison with commercially available samples (Supplementary Materials IV).

## Figures and Tables

**Table 1:**
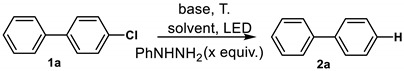
The evaluation of various reaction conditions ^a^.

Entry	x	Light Source	T (°C)	Base	Solvent	Yield (%) ^b^
1	0	365 nm	50	4eq. *t-*BuOK + 8eq. Et_3_N	DMF	6
2	1	365 nm	50	4eq. *t-*BuOK + 8eq. Et_3_N	DMF	13
3	2	365 nm	50	4eq. *t-*BuOK + 8eq. Et_3_N	DMF	29
4	4	365 nm	50	4eq. *t-*BuOK + 8eq. Et_3_N	DMF	83
5	6	365 nm	50	4eq. *t-*BuOK + 8eq. Et_3_N	DMF	72
6	4	365 nm	50	4eq. *t-*BuOK + 8eq. Et_3_N	MeCN	38
7	4	365 nm	50	4eq. *t-*BuOK + 8eq. Et_3_N	1,4-Dioxane	15
8	4	365 nm	50	4eq. *t-*BuOK + 8eq. Et_3_N	*t*-BuOH	19
9	4	254 nm	50	4eq. *t-*BuOK + 8eq. Et_3_N	DMF	21
10	4	395 nm	50	4eq. *t-*BuOK + 8eq. Et_3_N	DMF	42
11	4	405 nm	50	4eq. *t-*BuOK + 8eq. Et_3_N	DMF	62
12	4	455 nm	50	4eq. *t-*BuOK + 8eq. Et_3_N	DMF	35
13	4	485 nm	50	4eq. *t-*BuOK + 8eq. Et_3_N	DMF	38
14	4	Dark	50	4eq. *t-*BuOK + 8eq. Et_3_N	DMF	7
15	4	365 nm	50	4eq. *t-*BuOK	DMF	26
16	4	365 nm	50	8eq. Et_3_N	DMF	8
17	4	365 nm	50	8eq. *t-*BuOK + 16eq. Et_3_N	DMF	88
18	4	365 nm	50	8eq. *t-*BuOK + 16eq. *N,N,N*′*,N*′-Tetramethylethylenediamine	DMF	92
***19***	***4***	***365 nm***	***50***	***8eq. t-BuOK + 16eq. N,N***′***-Dimethyl-1,2-ethanediamine***	***DMF***	***95***
20	4	365 nm	35	8eq. *t-*BuOK + 16eq. *N,N*′-Dimethyl-1,2-ethanediamine	DMF	42

^a^ General conditions: 1a (0.2 mmol), base, and 3 mL solvent at the reaction temperature for 48 h in a 35 mL sealed tube. ^b^ Yields were determined via HPLC, using 4-methylbiphenyl as an internal standard.

## Data Availability

Not applicable.

## Supplementary Materials

The following supporting information can be downloaded at: https://www.mdpi.com/article/10.3390/molecules28196915/s1. Supplementary Materials I: General Information; Supplementary Materials II: Procedure for the Dichlorination of 4-chloro-1,1′-biphenyl; Supplementary Materials III: General Procedure for the Reduction of C–X Bond; Supplementary Materials IV: Spectra of ^1^H NMR and ^13^C NMR.

## Sample Availability

Available.
