# Supplementary material for: Photo-Induced, Phenylhydrazine-Promoted Transition-Metal-Free Dehalogenation of Aryl Fluorides, Chlorides, Bromides, and Iodides
Source: Molecules. 2023 Oct 3;28(19):6915. doi: 10.3390/molecules28196915 (PMC10574415; doi:10.3390/molecules28196915)
Supplement: Supplementary file 1 [file molecules-28-06915-s001.zip › molecules-2636975-Supplementary.pdf]

Photo-Induced, Phenylhydrazine-Promoted Transition-Metal-Free Dehalogenation of Aryl Fluorides, Chlorides, Bromides, and Iodides

Yiwei Zhu*, Zhimin Wu, Hongcai Sun, Junjun Ding

School of Chemistry and Environmental Engineering, Anhui Polytechnic University, Wuhu 241000, Chi-na; dingjunjun@nexchip.com.cn

* Correspondence: yiwei.zhu@ahpu.edu.cn

Contents

I. General Information S1
II. Procedure for the Dichlorination of 4-chloro-1,1'-biphenyl S2
III. General Procedure for the Reduction of C–X Bond S3
IV. Spectra of ^{1}H NMR and ^{13}C NMR S4

I. General Information

All reagents and solvents utilized in this study were procured from commercial sources and employed without further purification, unless otherwise specified. Nuclear magnetic resonance (NMR) spectra were acquired using Bruker AV300, Bruker AV400, and Bruker AV500M spectrometers, and chemical shifts were reported in parts per million (δ) relative to the internal standard, tetramethylsilane (TMS), positioned at 0 ppm in $CDCl_3$. The determinations of some chemicals were compared with the spectra reported in the literature. The light sources employed were LED lamps with wavelengths ranging from 254 nm to 485 nm, providing an output power of 7W. The reaction progress was monitored via thin-layer chromatography (TLC). Column chromatography was performed using silica gel (200–300

mesh), and the compounds were visualized under ultraviolet light. Elution during column chromatography was achieved using a mixture of ethyl acetate and petroleum ether as the eluent. High-performance liquid chromatography (HPLC) analyses were conducted using a Shimadzu LC-16 spectrometer. Gas-chromatography–mass-spectrometry (GC-MS) analyses were carried out utilizing a Thermo TRACE 1300 ISQ LT spectrometer.

II. Procedure for the Deiodination of 4-chloro-1,1'-biphenyl

A 35 mL thick-walled pressure vessel equipped with a Teflon cap was charged with a magnetic stirring bar, 4-chloro-1,1'-biphenyl (0.2 mmol), t BuOK (1.6 mmol), $PhNHNH_2$ (0.8 mmol), N,N'-Dimethyl-1,2- ethanediamine (3.2 mmol), and dry DMF (3 mL). Subsequently, the pressure vessel was immersed in an oil bath along with LED lamps (365 nm, 7W × 4) at a temperature of 50 °C. To ensure light exclusion, the entire reaction system was carefully shielded with tinfoil film. The reaction mixture was stirred for 48 hours. Following the designated reaction time, 0.2 mmol of 4-methylbiphenyl was introduced into the pressure vessel. Subsequently, 10 mL of water was added to terminate the reaction, and the resulting mixture was extracted using ethyl acetate (5 mL × 4). The combined organic extracts were washed with brine, dried with sodium sulfate, and then subjected to filtration for the subsequent HPLC analysis.

III. General Procedure for the Reduction of C−X Bond

A 35 mL thick-walled pressure vessel, equipped with a Teflon cap and a magnetic stirring bar, was charged with aryl halide (0.2 mmol), t-BuOK (1.6 mmol), $PhNHNH_2$ (0.8 mmol), N,N'-Dimethyl-1,2-ethanediamine (3.2 mmol), and dry DMF (3 mL). The pressure vessel was then positioned in an oil bath, accompanied by LED lamps emitting light at a wavelength of 365 nm and a power output of 7W × 4, while maintaining a temperature of 50 °C. To ensure light exclusion, the entire reaction system was meticulously wrapped with tinfoil film. The mixture was stirred for 48 or 96 hours, as specified. Following the designated reaction time, 10 mL of water was added to quench the reaction, and the resulting mixture was extracted using ethyl acetate (5 mL × 4). The combined organic extracts were subsequently washed with brine, dried with sodium sulfate, and concentrated under reduced pressure. The resulting residue was purified via preparative TLC on silica gel, using a gradient elution of petroleum ether and ethyl acetate (300:1–5:1) as the eluent, to yield the desired products. The confirmation of the products was accomplished via comparison with commercially available samples.

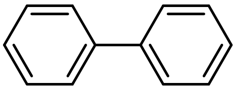

^{1}H NMR

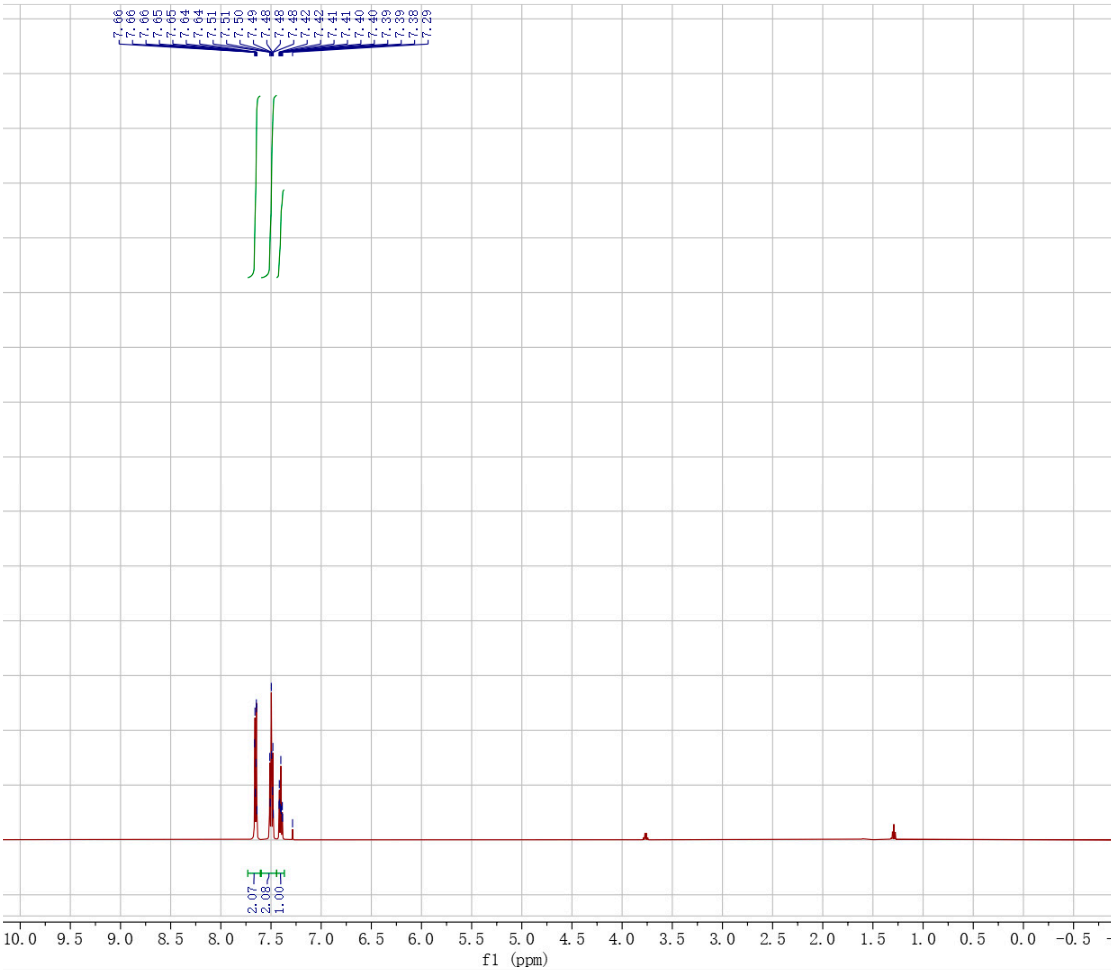
7.66 7.66 7.66 7.65 7.65 7.64 7.64 7.51 7.51 7.50 7.49 7.48 7.48 7.48 7.42 7.42 7.41 7.41 7.40 7.40 7.39 7.38 7.29
2.07
2.08
1.00
10.0 9.5 9.0 8.5 8.0 7.5 7.0 6.5 6.0 5.5 5.0 4.5 4.0 3.5 3.0 2.5 2.0 1.5 1.0 0.5 0.0 -0.5
f1 (ppm)

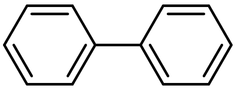

^{13}C NMR

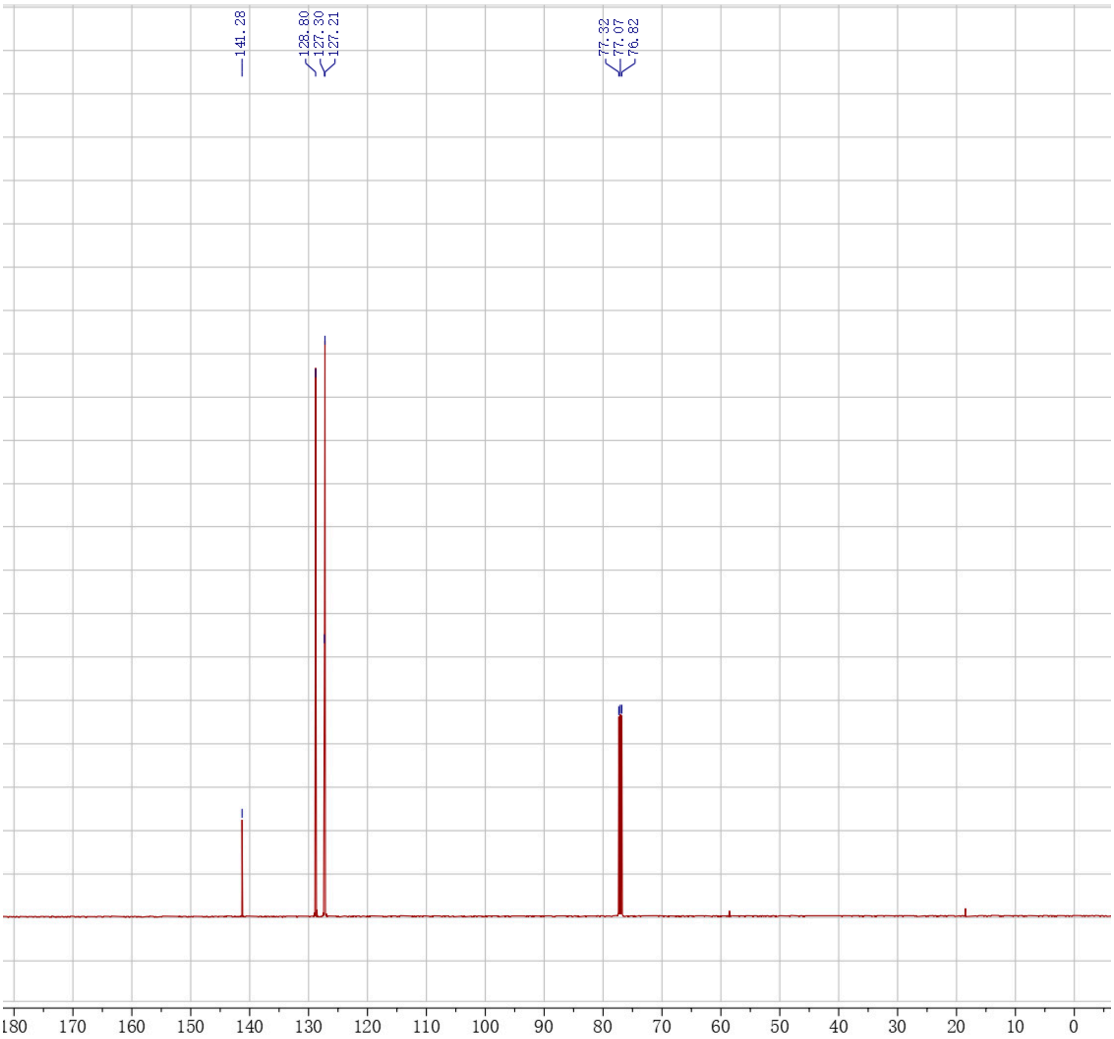
141.28
128.80
127.30
127.21
77.32
77.07
76.82
180 170 160 150 140 130 120 110 100 90 80 70 60 50 40 30 20 10 0

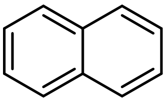

^{1}H NMR

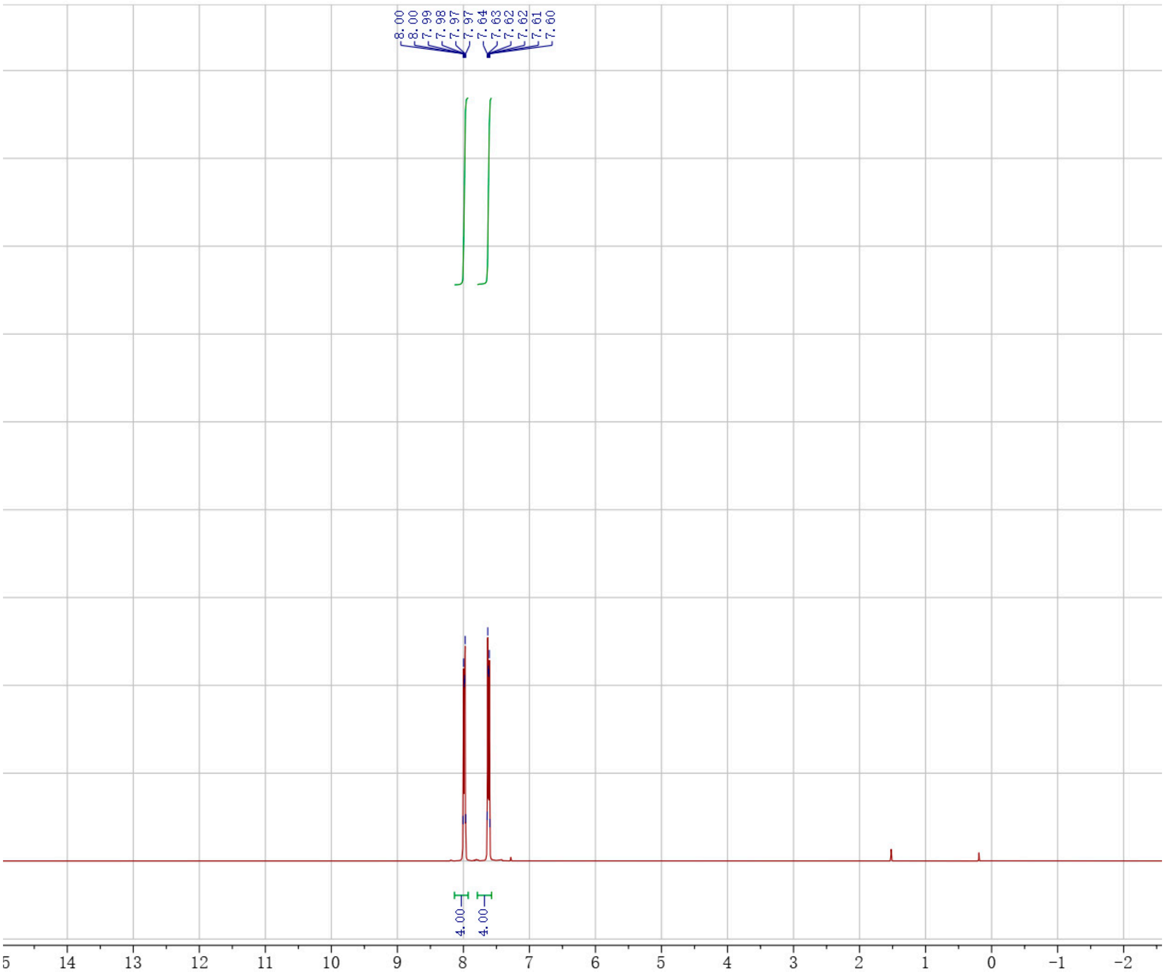

8.00
8.00
7.99
7.98
7.97
7.97
7.64
7.63
7.62
7.62
7.61
7.60
4.00
4.00
14
13
12
11
10
9
8
7
6
5
4
3
2
1
0
−1
−2

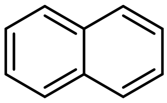

^{13}C NMR

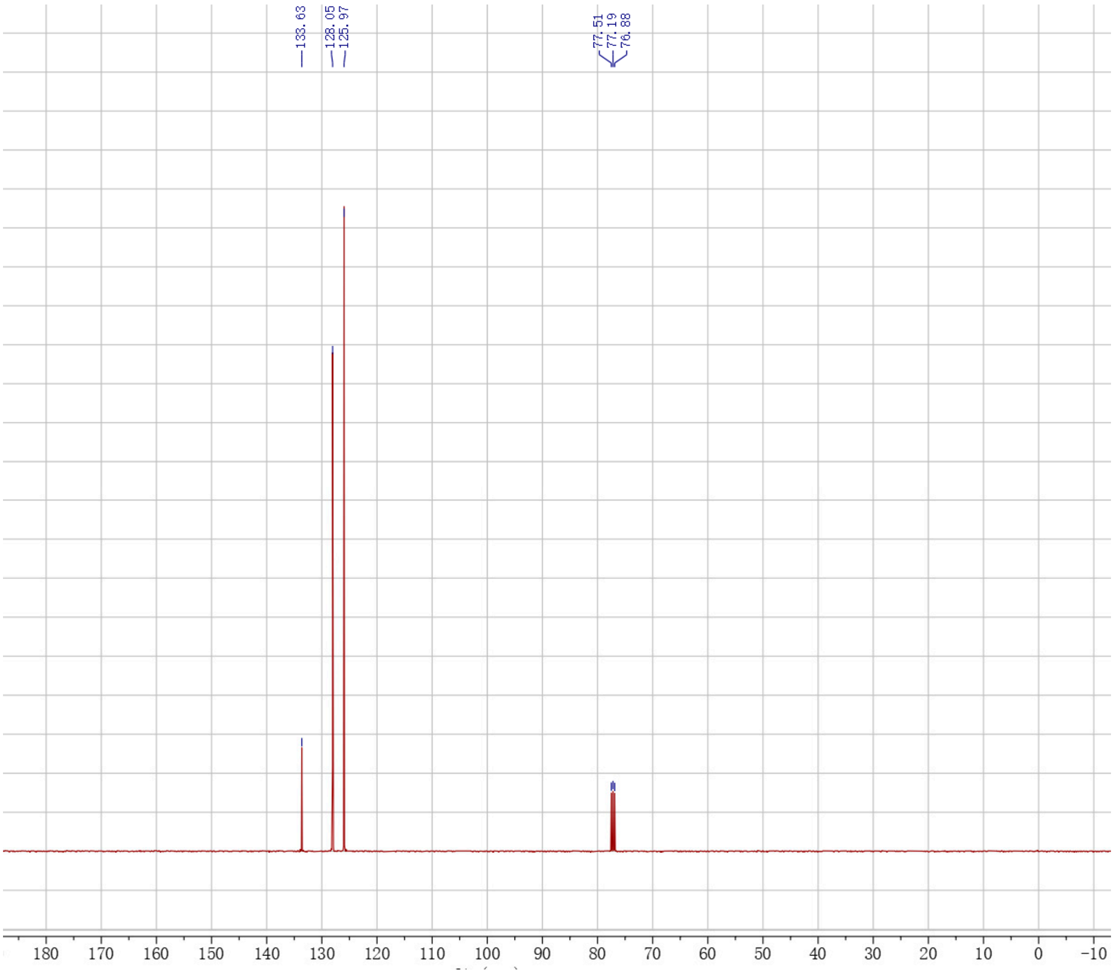
133.63
128.05
125.97
77.51
77.19
76.88
180 170 160 150 140 130 120 110 100 90 80 70 60 50 40 30 20 10 0 −10

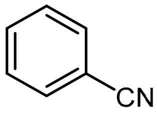

^{1}H NMR

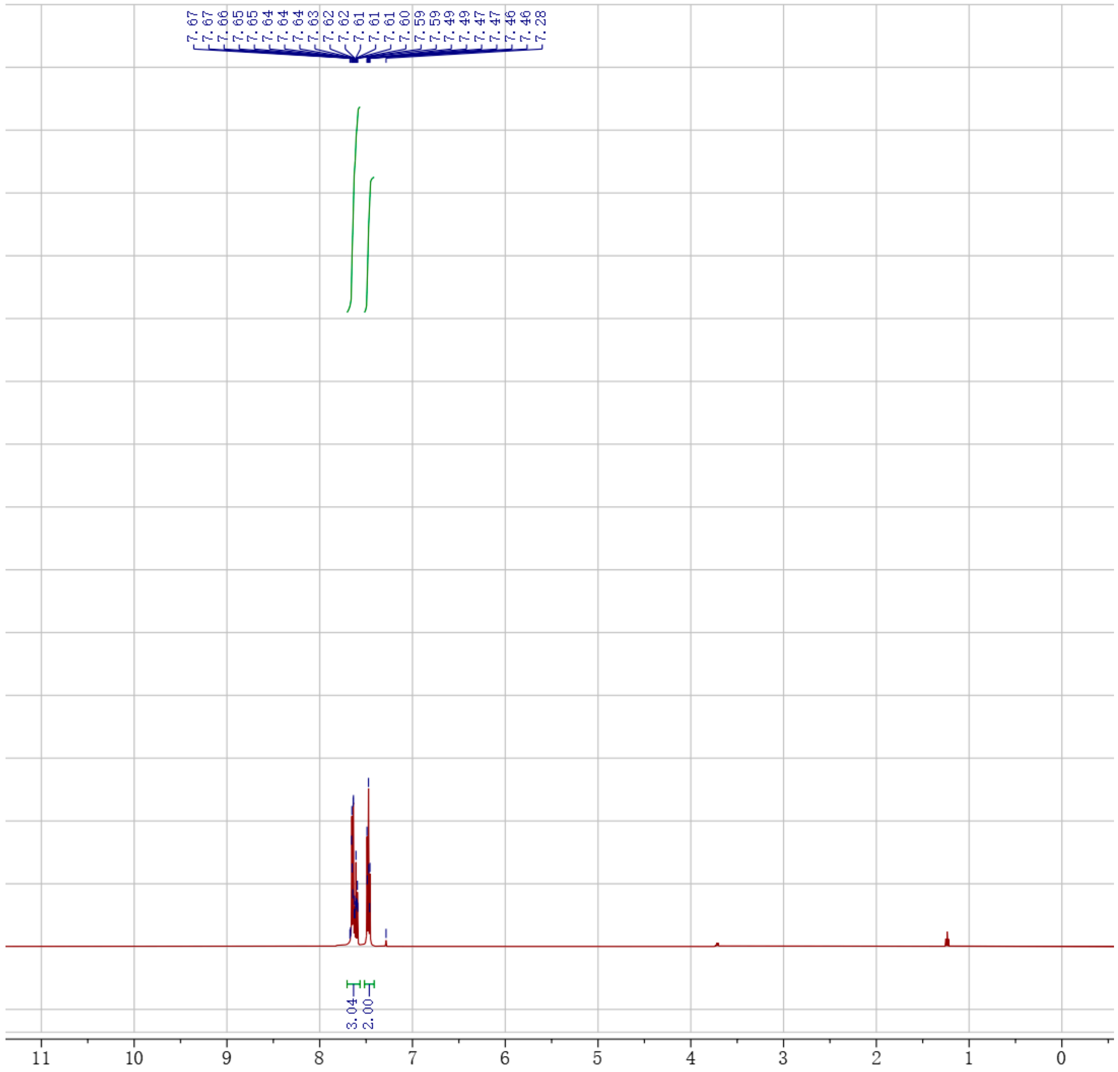

7.67
7.67
7.66
7.65
7.65
7.64
7.64
7.64
7.63
7.62
7.62
7.61
7.61
7.61
7.60
7.59
7.49
7.49
7.47
7.47
7.46
7.46
7.28
3.04
2.00
11
10
9
8
7
6
5
4
3
2
1
0

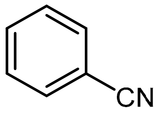
CN

^{13}C NMR

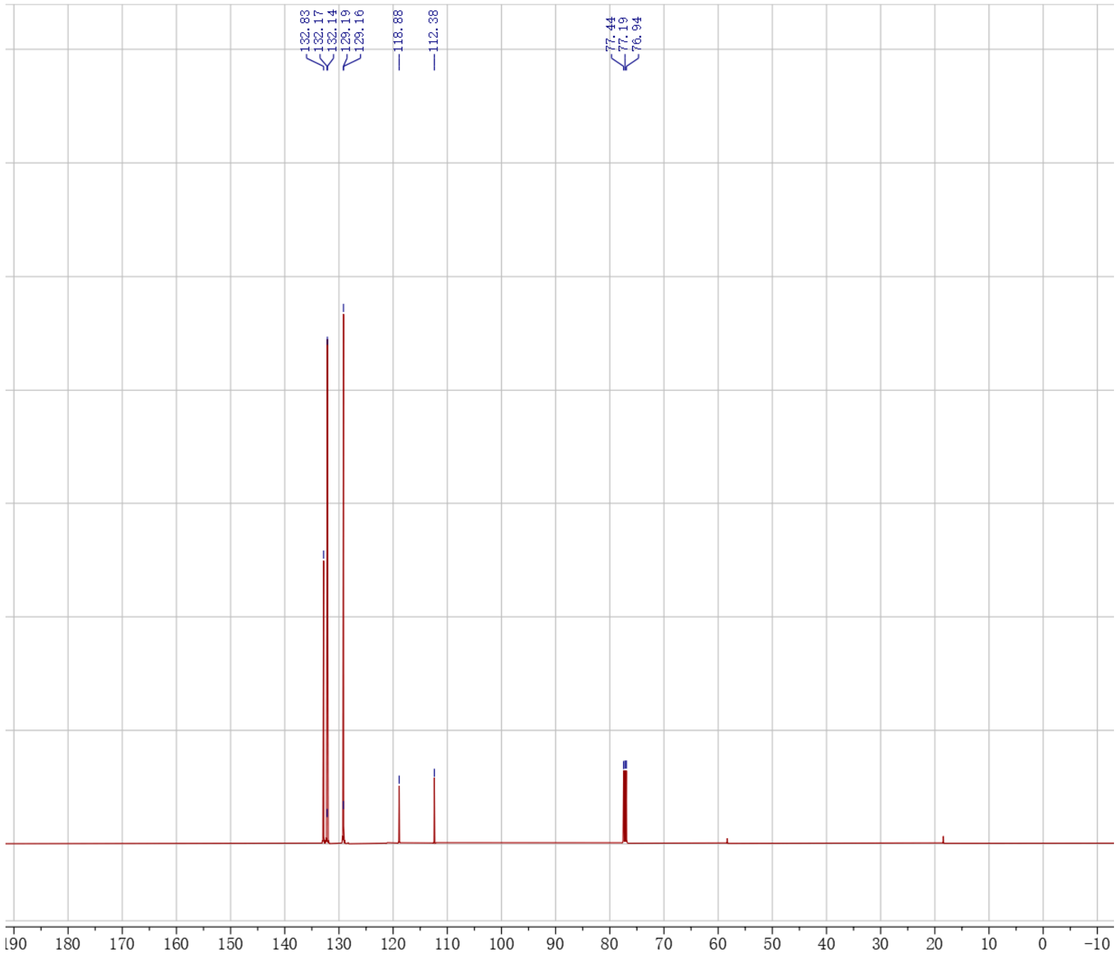
132.83
132.17
132.14
129.19
129.16
118.88
112.38
77.44
77.19
76.94
190 180 170 160 150 140 130 120 110 100 90 80 70 60 50 40 30 20 10 0 −10

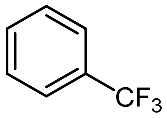
CF3

^{1}H NMR

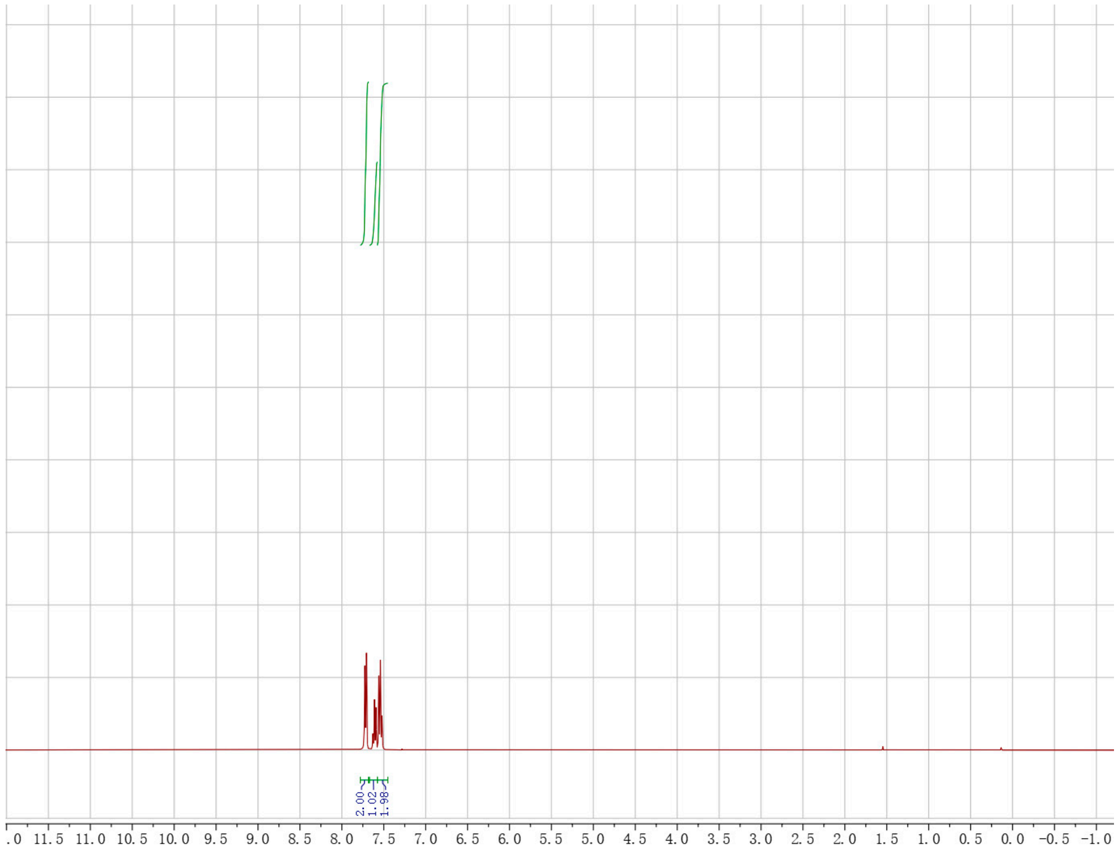
2.00
1.02
1.98
.0 11.5 11.0 10.5 10.0 9.5 9.0 8.5 8.0 7.5 7.0 6.5 6.0 5.5 5.0 4.5 4.0 3.5 3.0 2.5 2.0 1.5 1.0 0.5 0.0 -0.5 -1.0

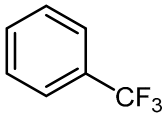
CF3

^{13}C NMR

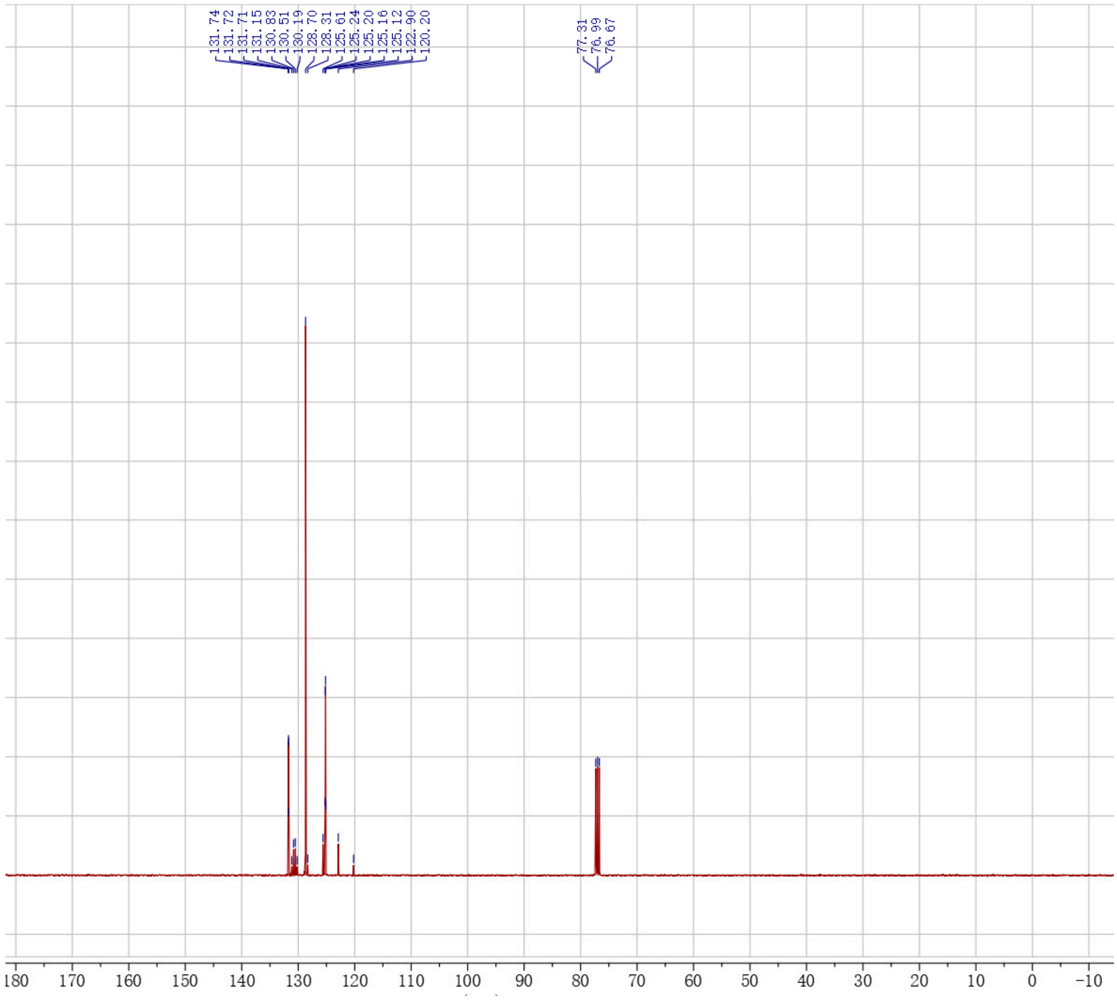
131.74
131.72
131.71
131.15
130.83
130.51
130.19
128.70
128.31
125.61
125.24
125.20
125.16
125.12
122.90
120.20
77.31
76.99
76.67
180
170
160
150
140
130
120
110
100
90
80
70
60
50
40
30
20
10
0
−10

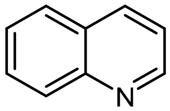
N

^{1}H NMR

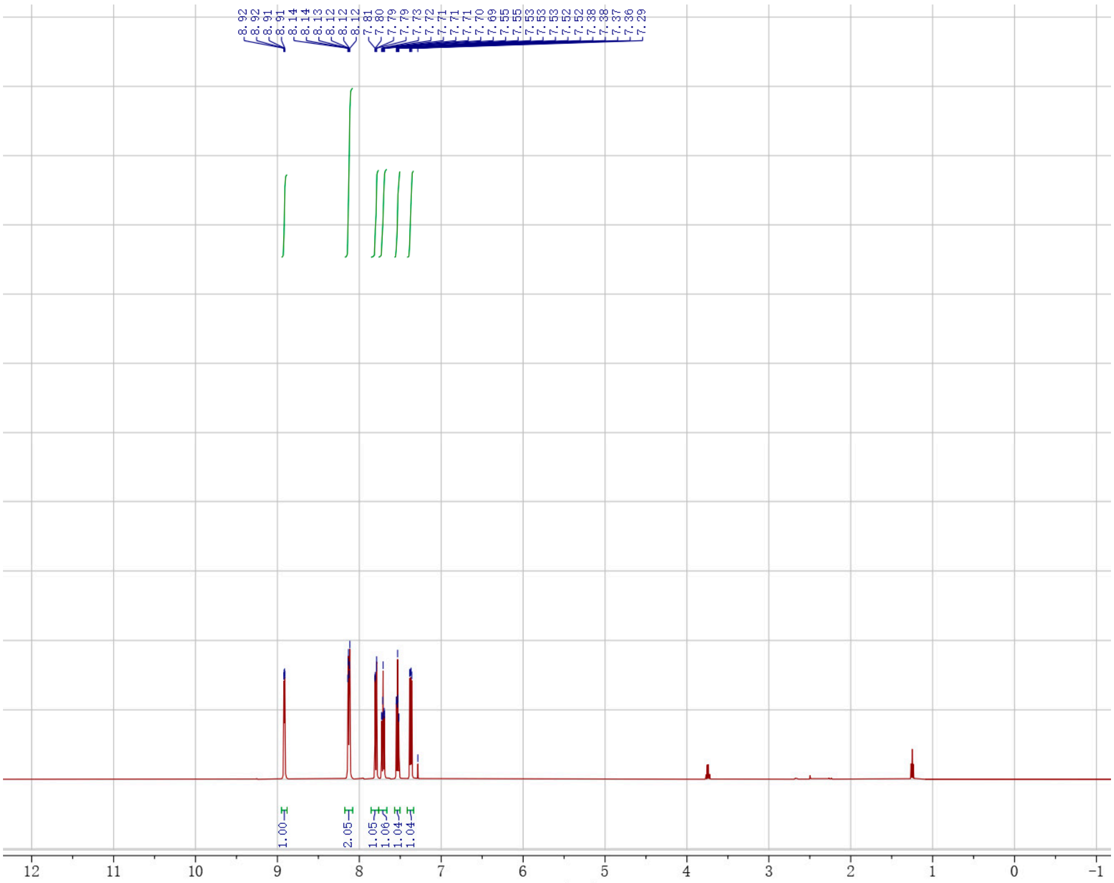
8.92
8.92
8.91
8.91
8.14
8.14
8.13
8.12
8.12
8.12
7.81
7.80
7.79
7.79
7.73
7.72
7.71
7.71
7.71
7.70
7.69
7.55
7.55
7.53
7.53
7.53
7.52
7.52
7.38
7.38
7.37
7.36
7.29
1.00
2.05
1.05
1.06
1.04
1.04
12
11
10
9
8
7
6
5
4
3
2
1
0
−1

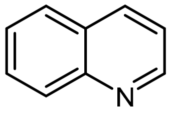

^{13}C NMR

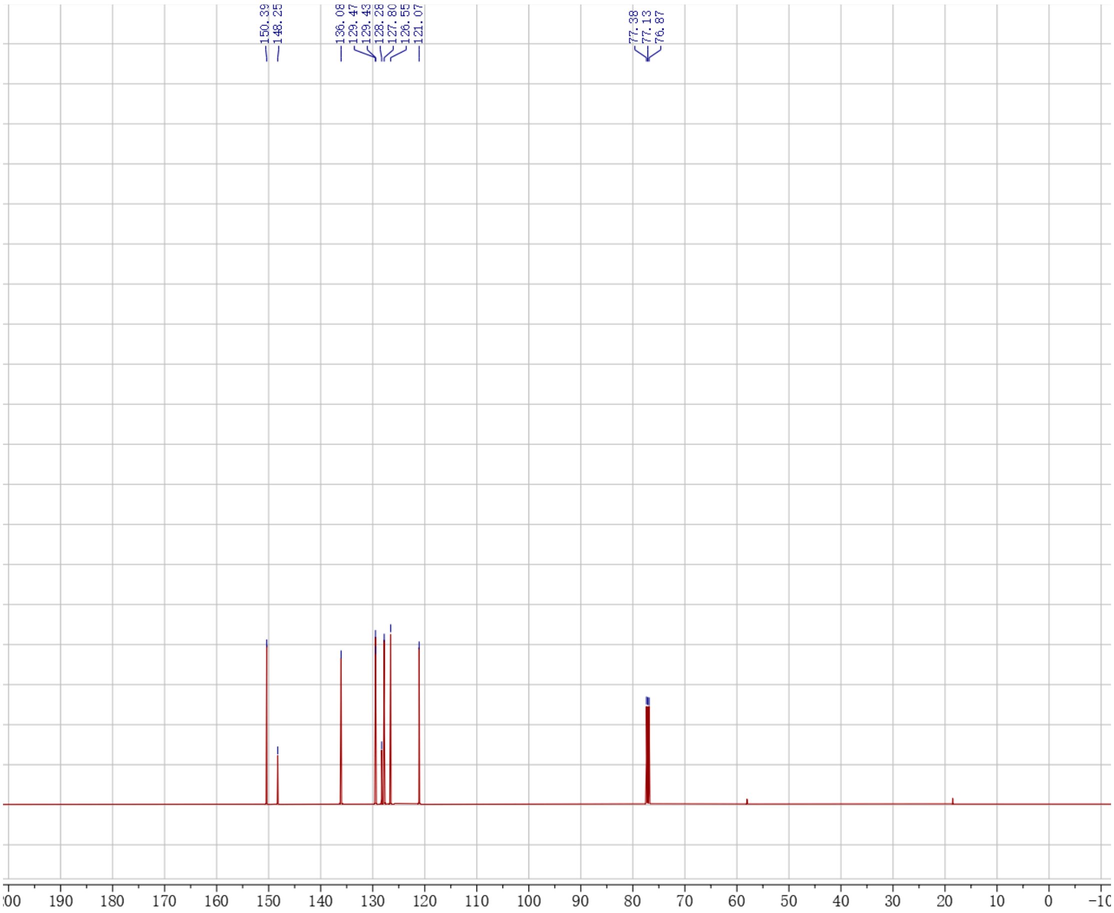
150.39
148.25
136.08
129.47
129.43
128.28
127.80
126.55
121.07
77.38
77.13
76.87
200 190 180 170 160 150 140 130 120 110 100 90 80 70 60 50 40 30 20 10 0 -10

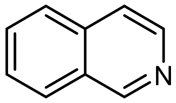

^{1}H NMR

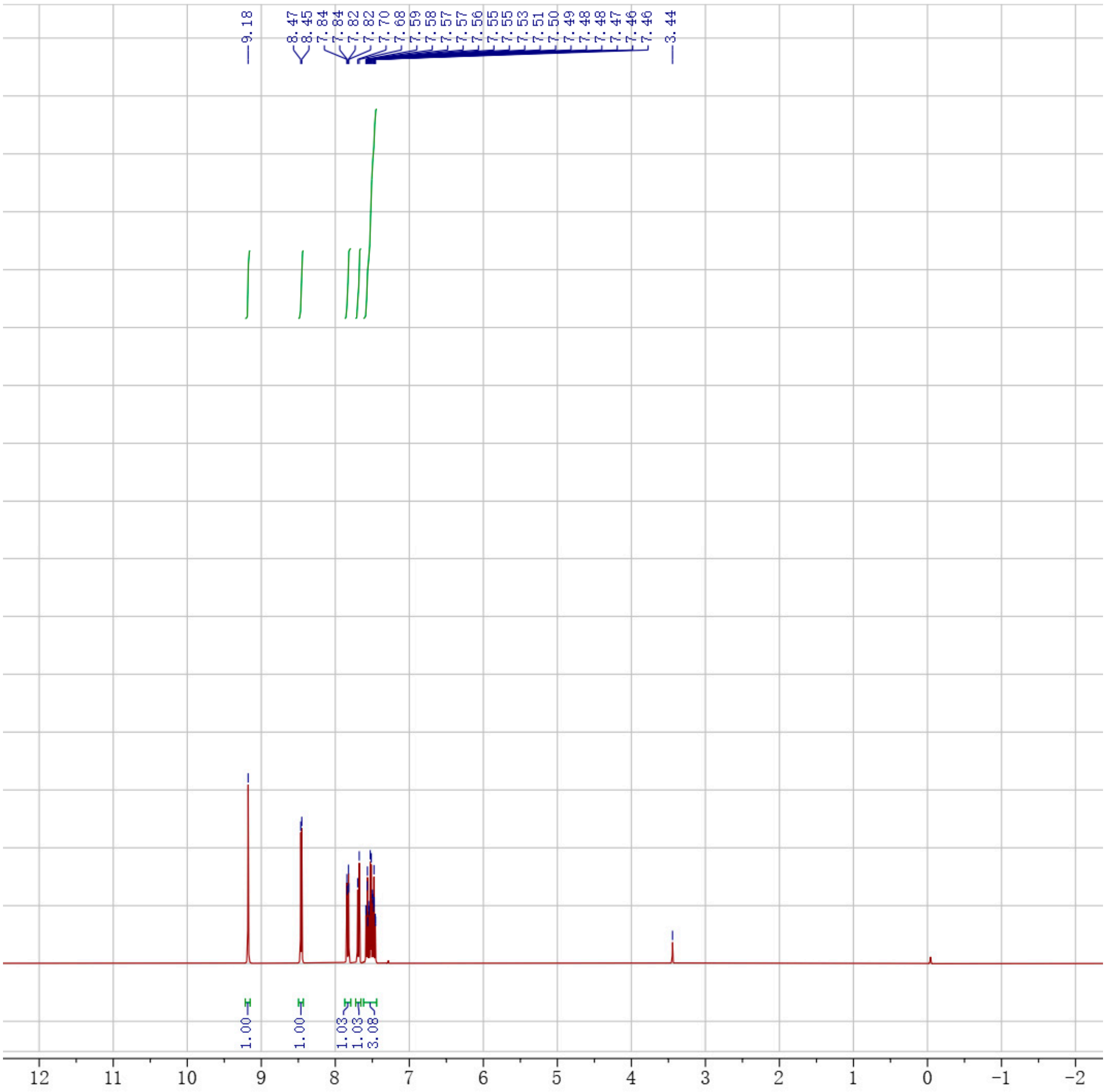
9.18
8.47
8.45
7.84
7.84
7.82
7.82
7.70
7.68
7.59
7.58
7.57
7.57
7.56
7.55
7.55
7.53
7.51
7.50
7.49
7.48
7.48
7.47
7.46
7.46
3.44
1.00
1.00
1.03
1.03
3.08
12
11
10
9
8
7
6
5
4
3
2
1
0
−1
−2

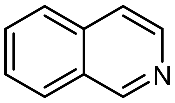

^{13}C NMR

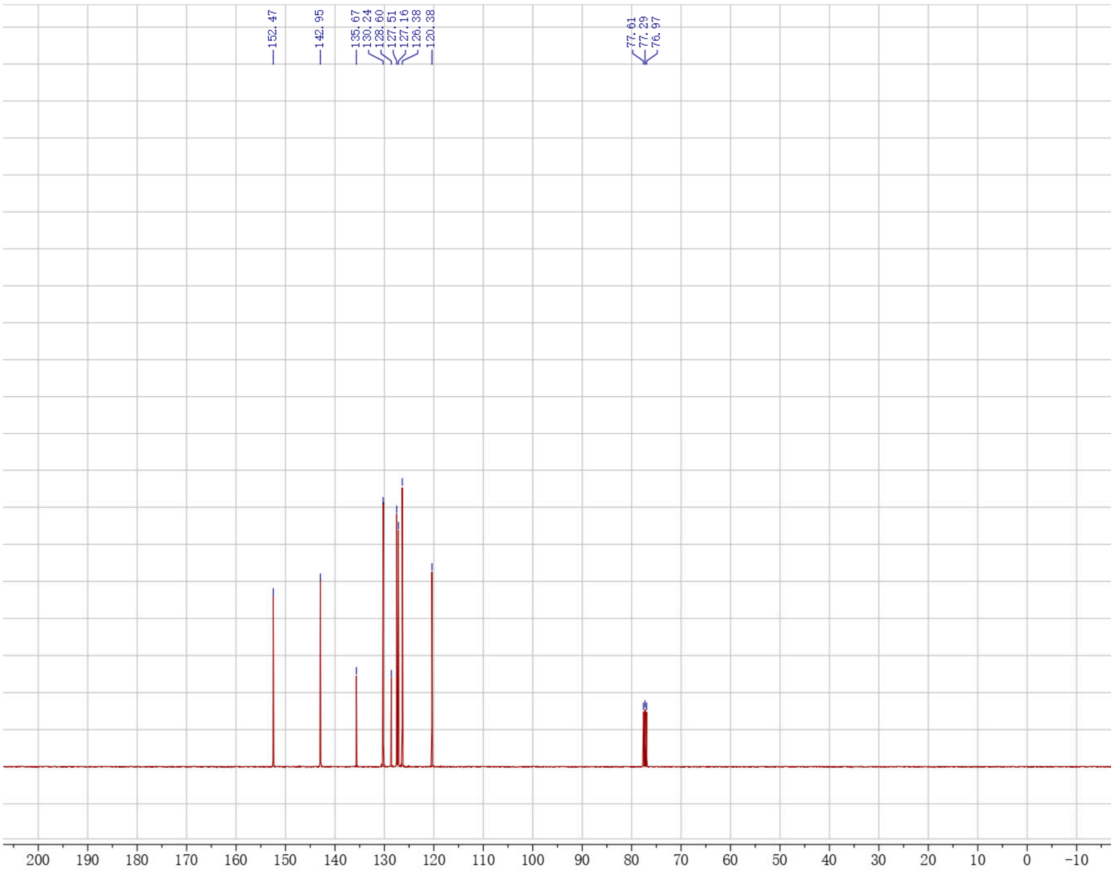

152.47
142.95
135.67
130.24
128.60
127.51
127.16
126.38
120.38
77.61
77.29
76.97
200
190
180
170
160
150
140
130
120
110
100
90
80
70
60
50
40
30
20
10
0
−10

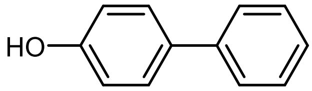

^{1}H NMR

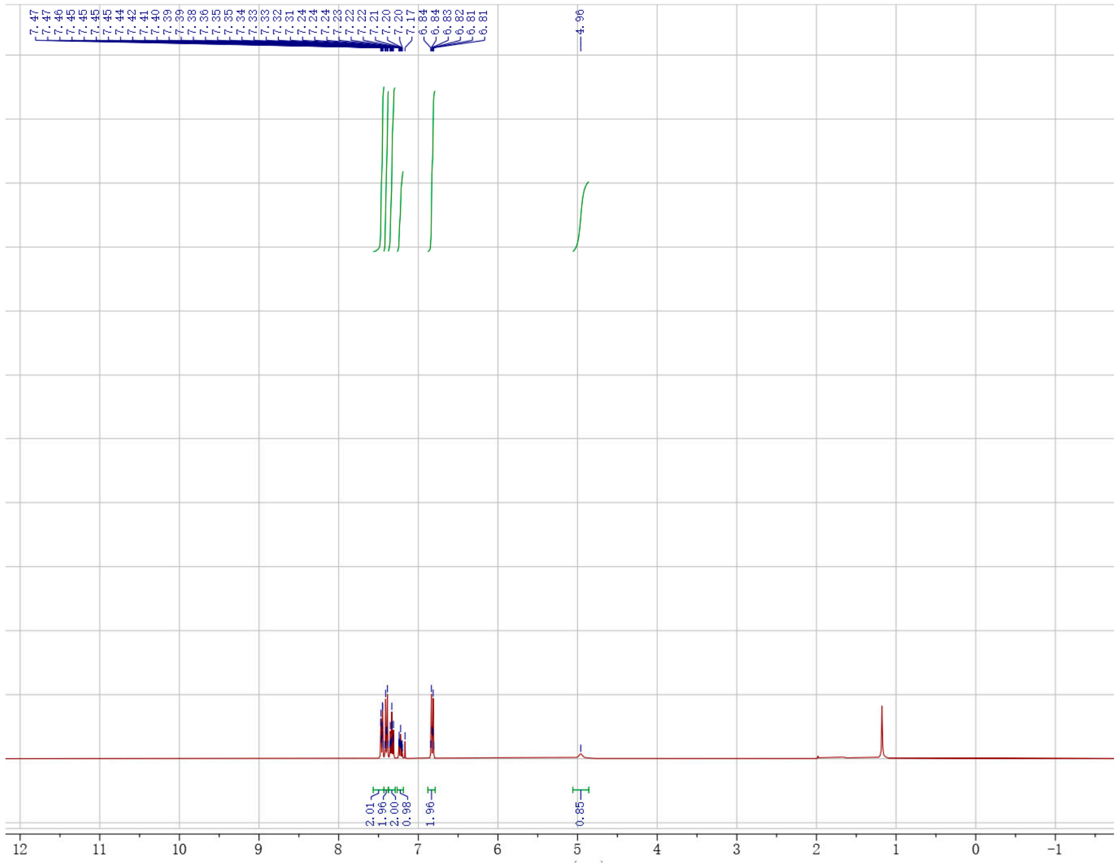

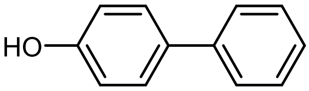
HO

^{13}C NMR

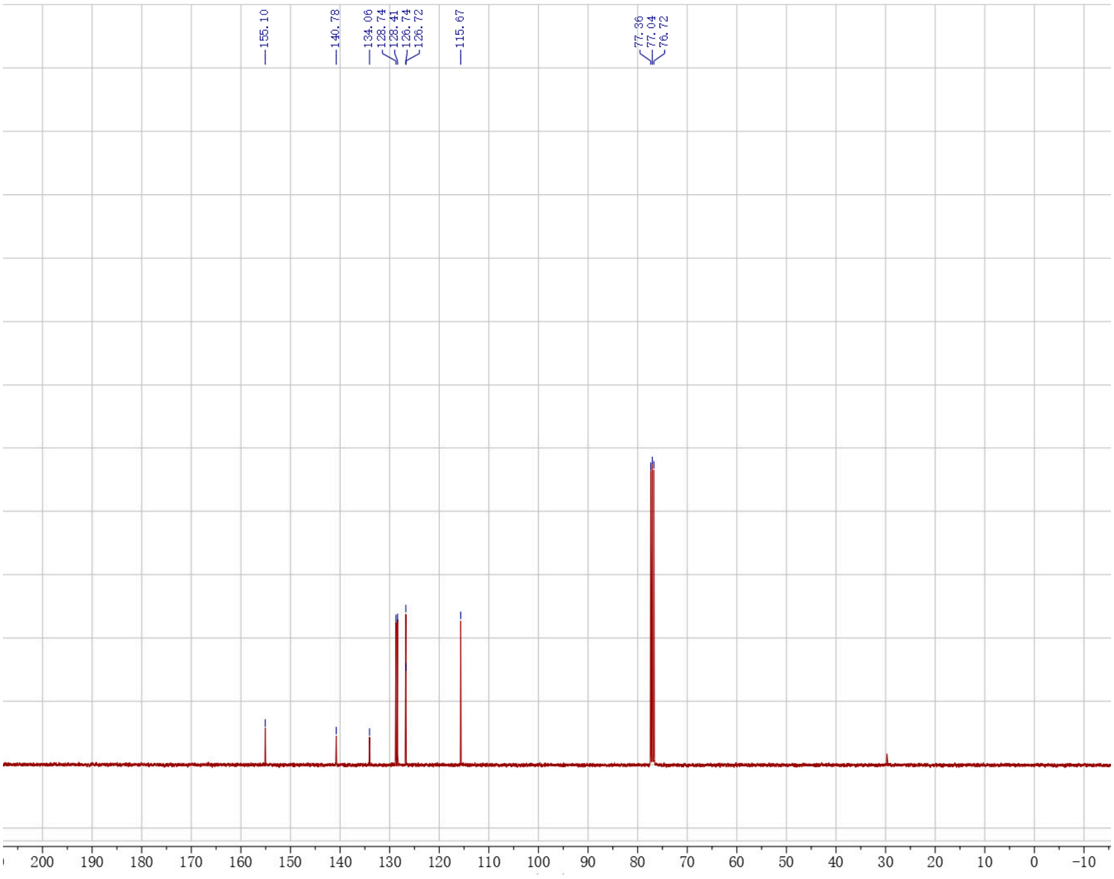
155.10
140.78
134.06
128.74
128.41
126.74
126.72
115.67
77.36
77.04
76.72
200 190 180 170 160 150 140 130 120 110 100 90 80 70 60 50 40 30 20 10 0 −10

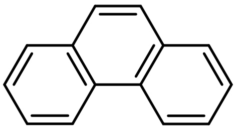

^{1}H NMR

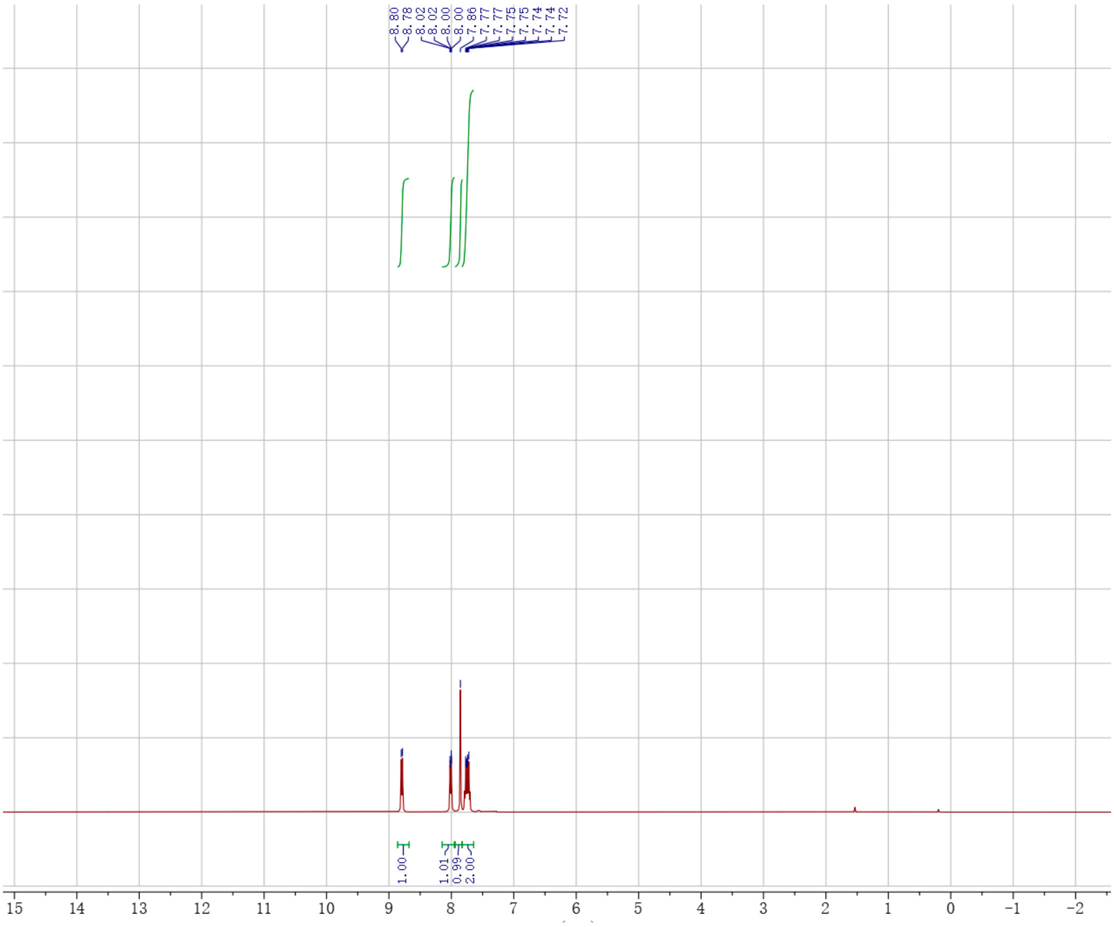

8.80
8.78
8.02
8.02
8.00
8.00
7.86
7.77
7.77
7.75
7.75
7.74
7.72
1.00
1.01
0.99
2.00
15
14
13
12
11
10
9
8
7
6
5
4
3
2
1
0
−1
−2

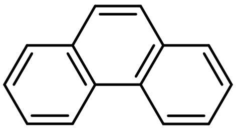

^{13}C NMR

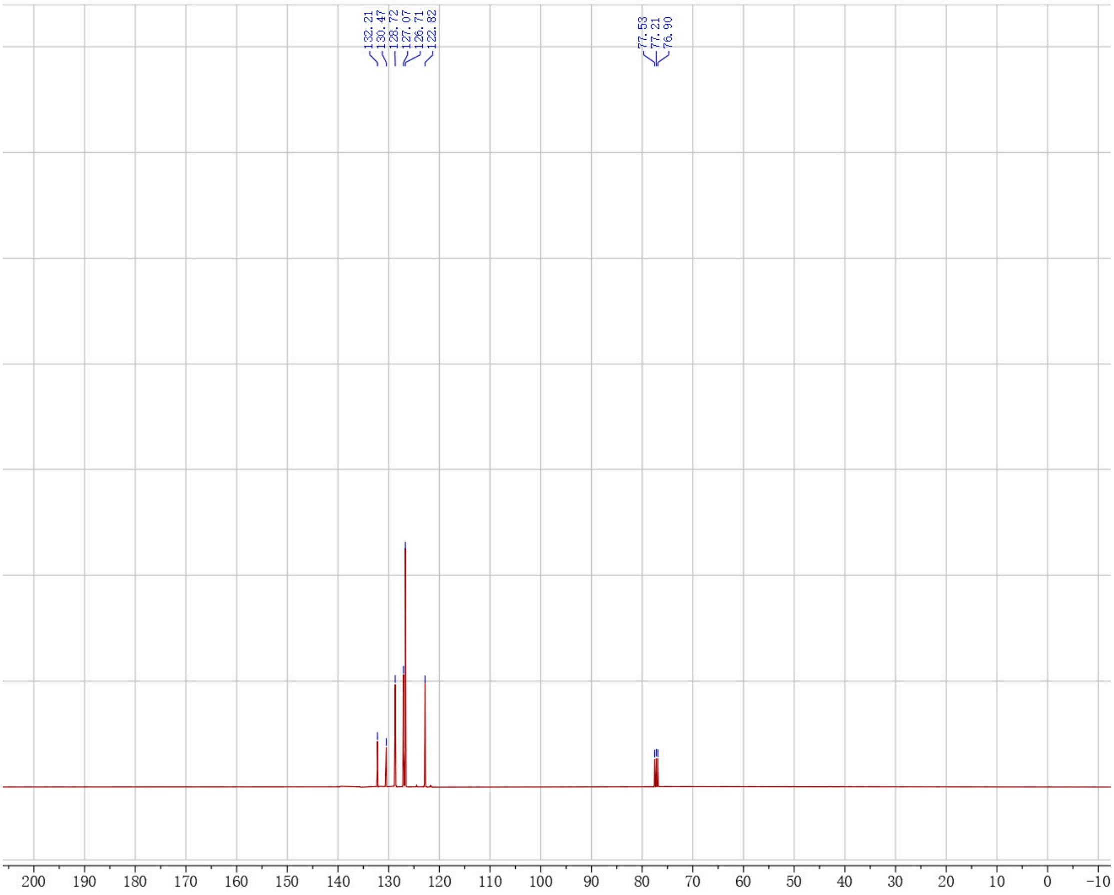

132.21
130.47
128.72
127.07
126.71
122.82
77.53
77.21
76.90
200
190
180
170
160
150
140
130
120
110
100
90
80
70
60
50
40
30
20
10
0
−10

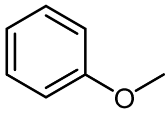

^{1}H NMR

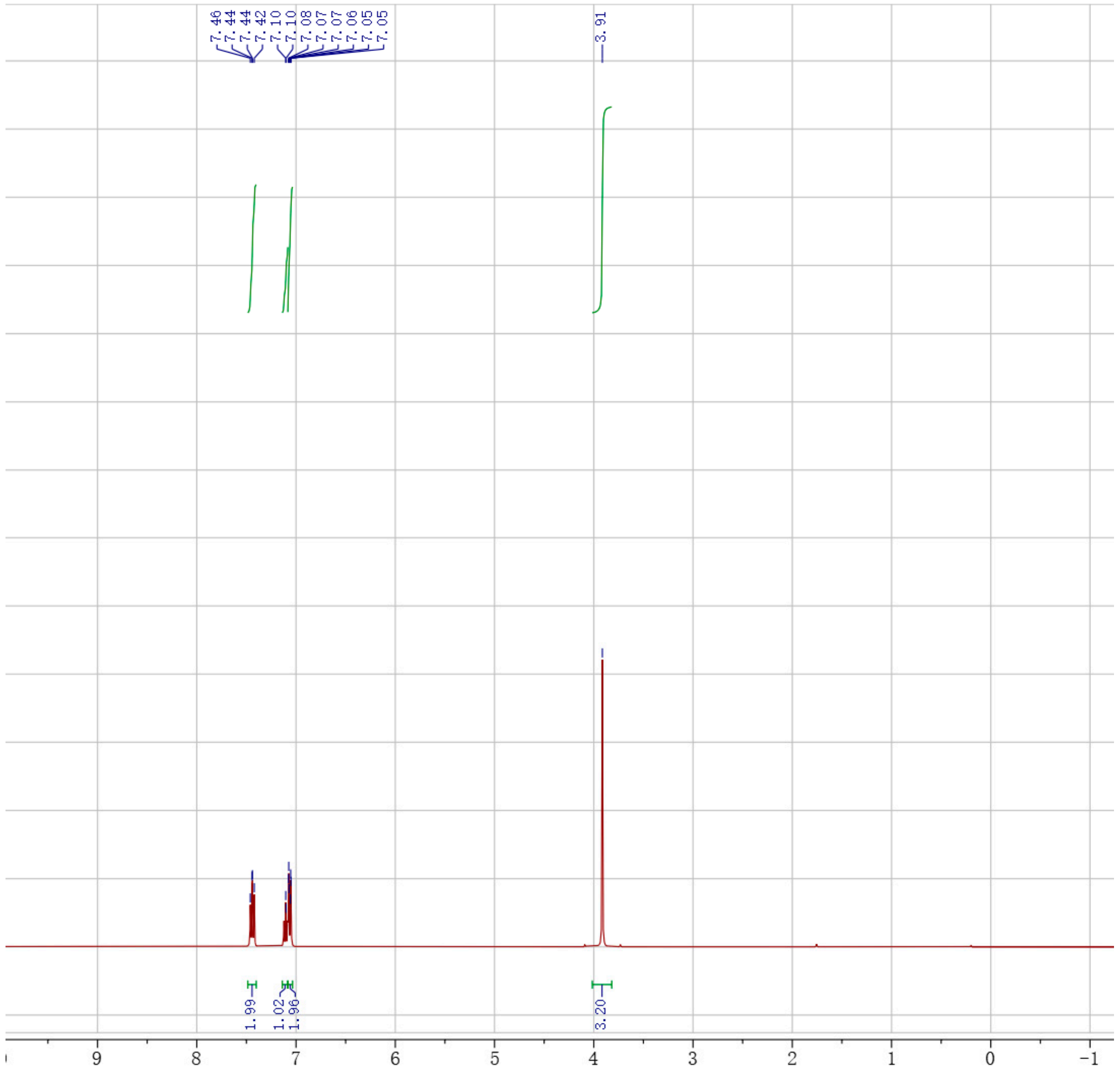
7.46
7.44
7.44
7.42
7.10
7.10
7.08
7.07
7.07
7.06
7.05
7.05
3.91
1.99
1.02
1.96
3.20
9
8
7
6
5
4
3
2
1
0
−1

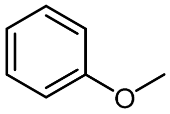

^{13}C NMR

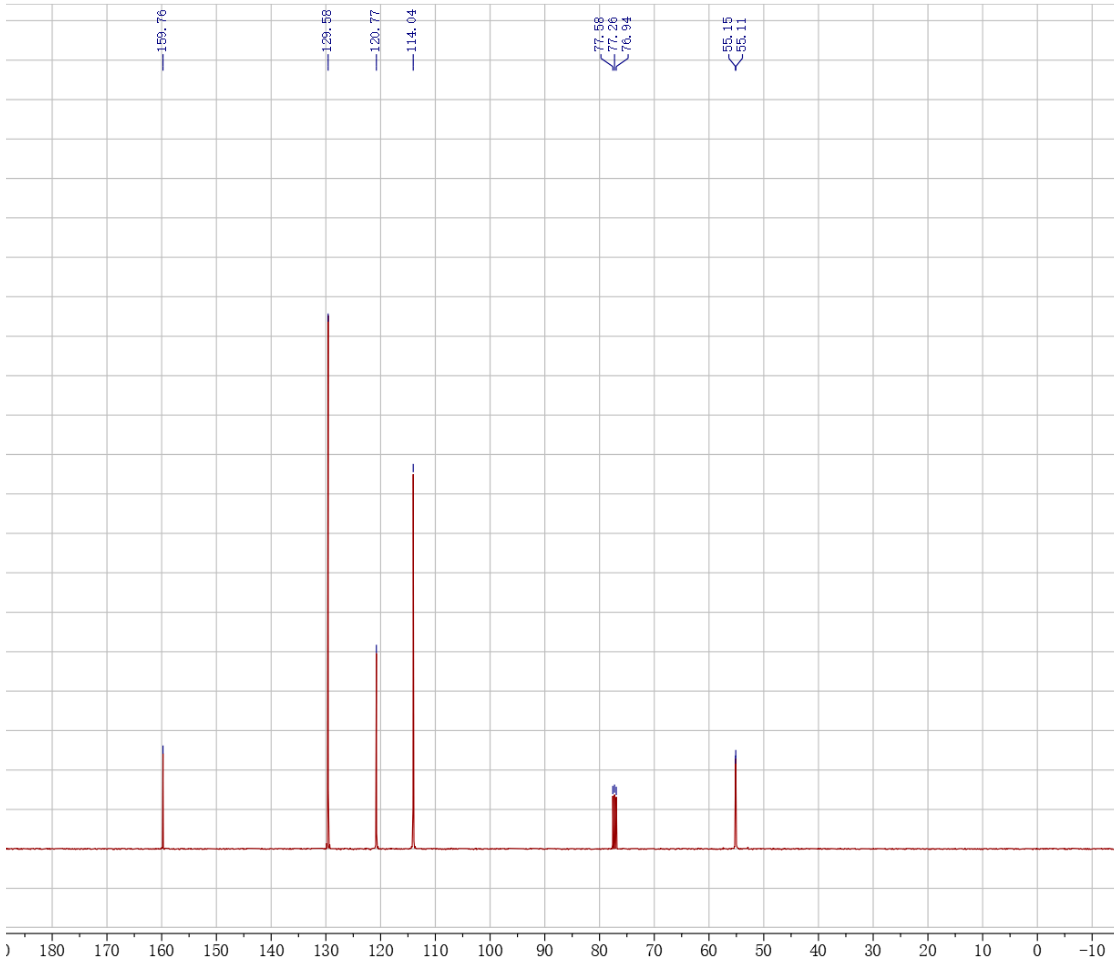

159.76
129.58
120.77
114.04
77.58
77.26
76.94
55.15
55.11
180 170 160 150 140 130 120 110 100 90 80 70 60 50 40 30 20 10 0 −10

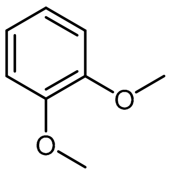

^{1}H NMR

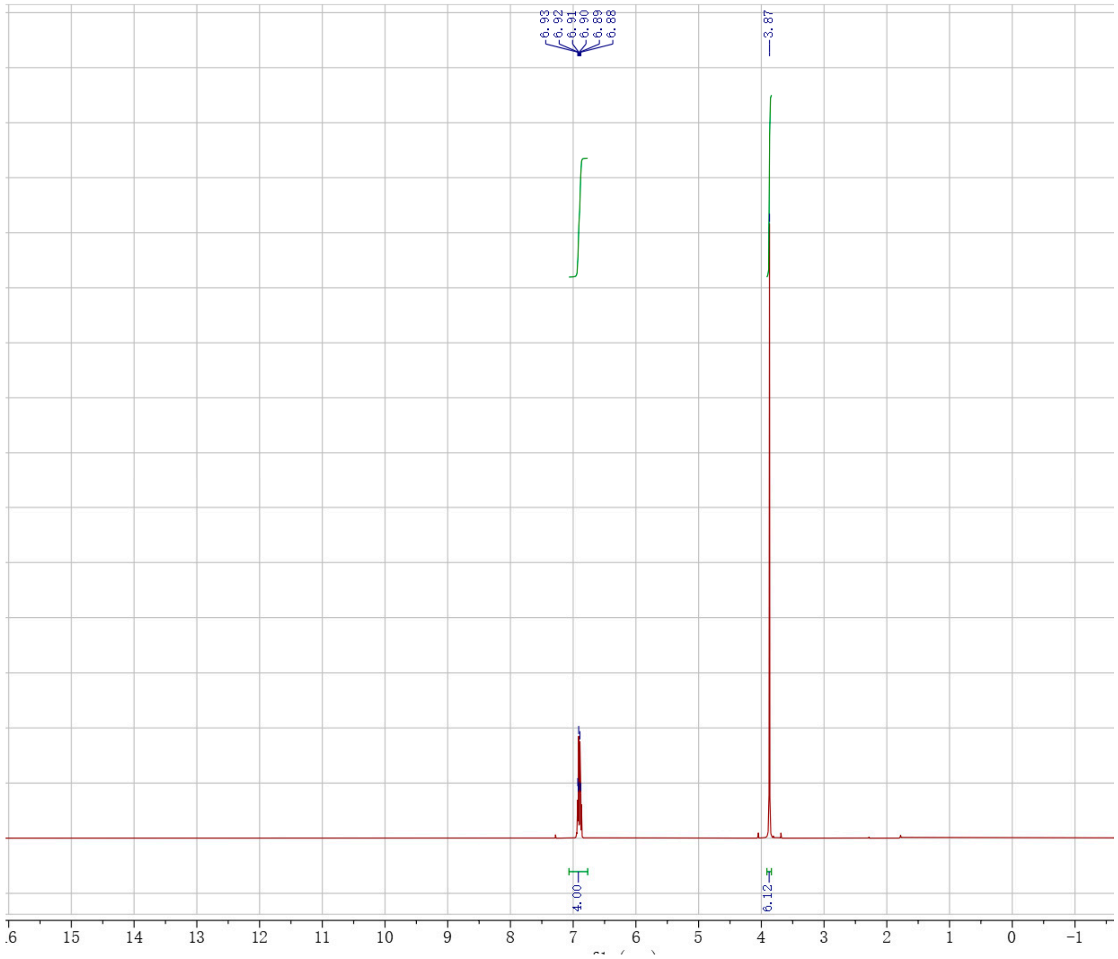

6.93
6.92
6.91
6.90
6.89
6.88
3.87
4.00
6.12
15
14
13
12
11
10
9
8
7
6
5
4
3
2
1
0
-1

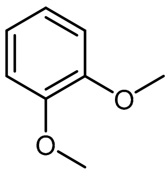

^{13}C NMR

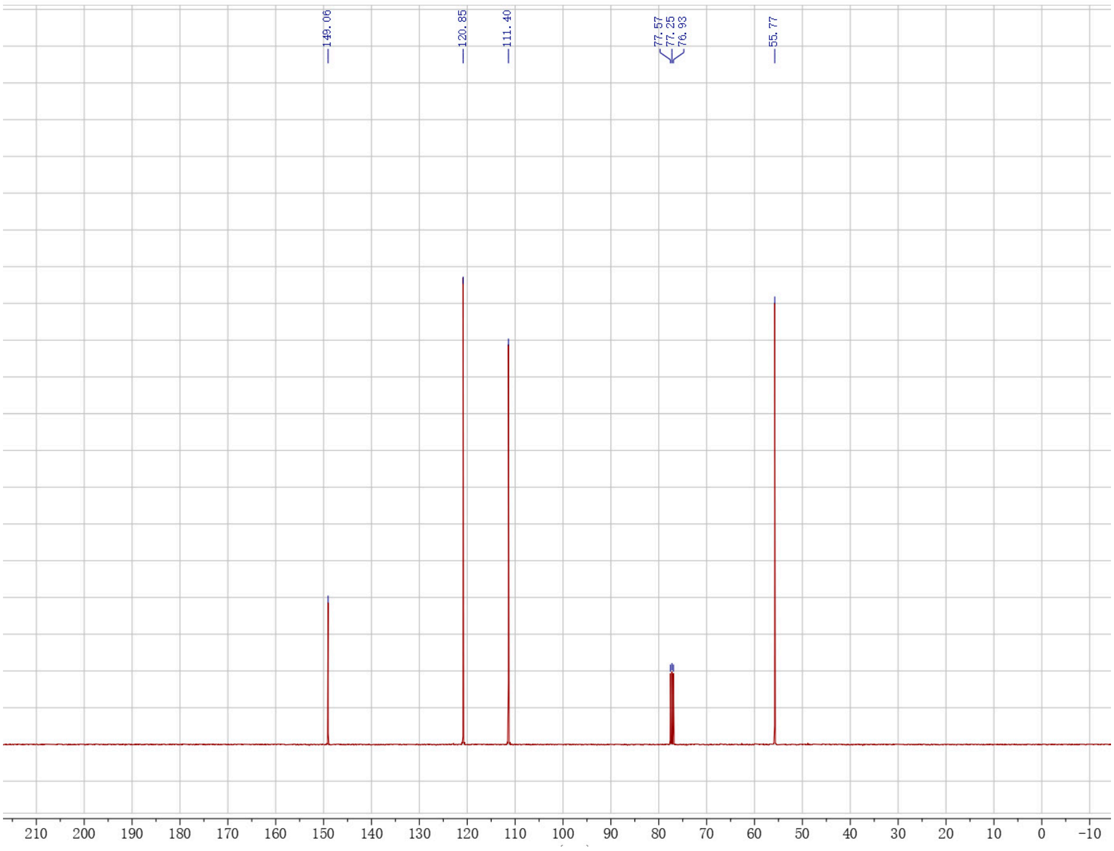
149.06
120.85
111.40
77.57
77.25
76.93
55.77
210 200 190 180 170 160 150 140 130 120 110 100 90 80 70 60 50 40 30 20 10 0 -10

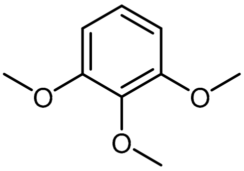

^{1}H NMR

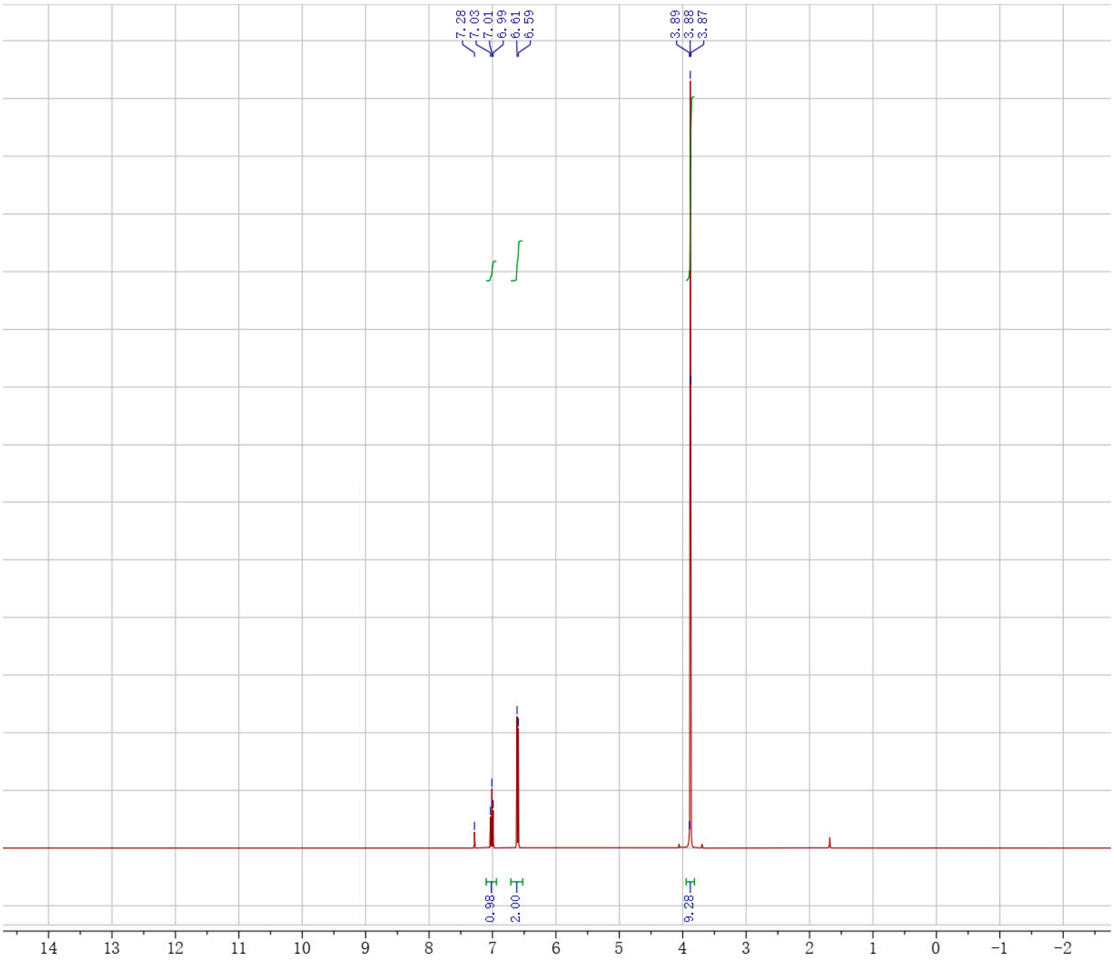
7.28
7.03
7.01
6.99
6.61
6.59
3.89
3.88
3.87
0.98
2.00
9.28
14
13
12
11
10
9
8
7
6
5
4
3
2
1
0
−1
−2

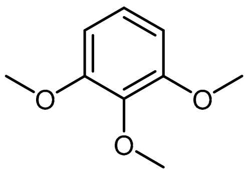

^{13}C NMR

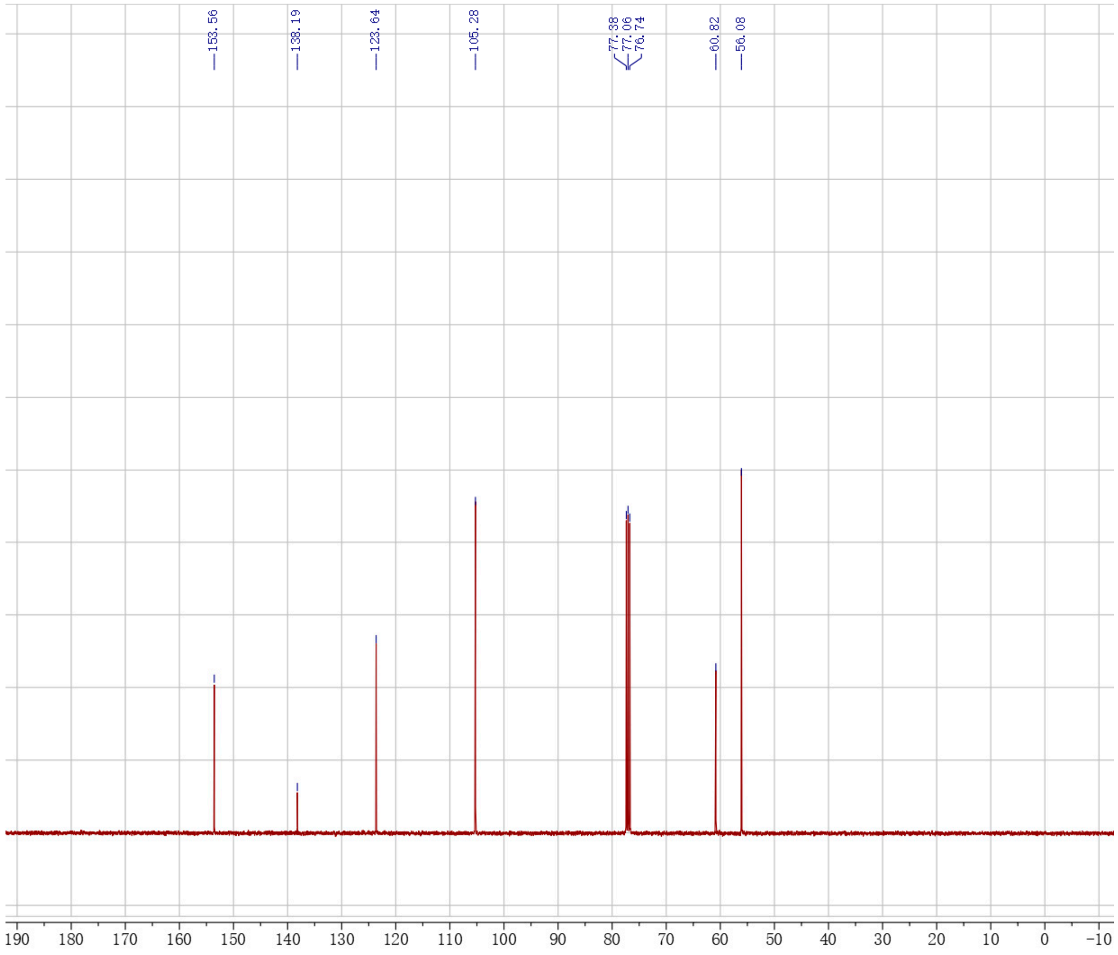
153.56
138.19
123.64
105.28
77.38
77.06
76.74
60.82
56.08
190
180
170
160
150
140
130
120
110
100
90
80
70
60
50
40
30
20
10
0
−10

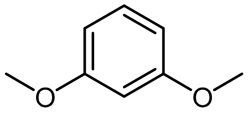

^{1}H NMR

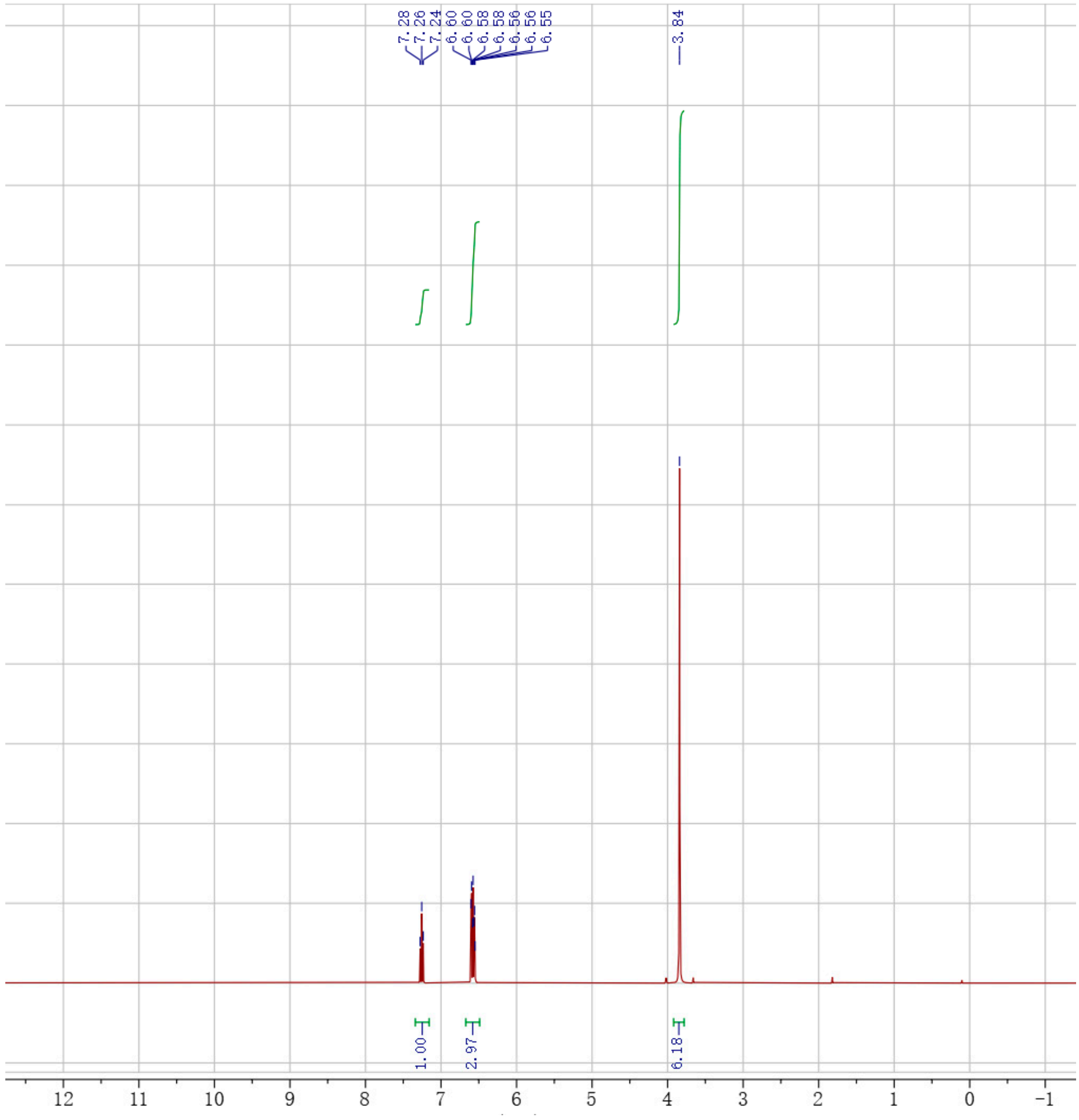

7.28
7.26
7.24
6.60
6.60
6.58
6.58
6.56
6.56
6.55
3.84
1.00
2.97
6.18
12 11 10 9 8 7 6 5 4 3 2 1 0 −1

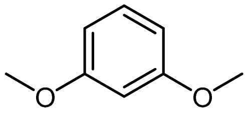

^{13}C NMR

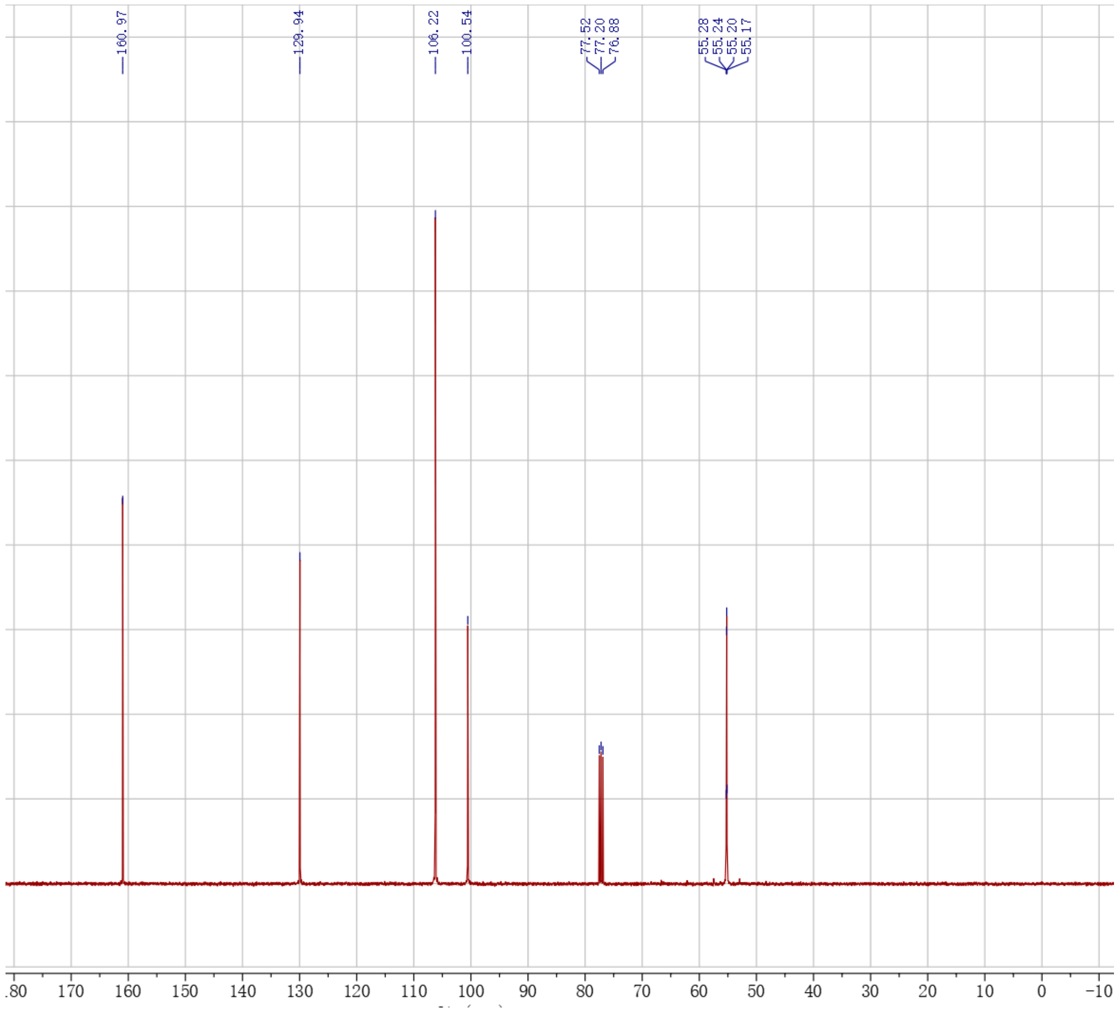

160.97
129.94
106.22
100.54
77.52
77.20
76.88
55.28
55.24
55.20
55.17
180
170
160
150
140
130
120
110
100
90
80
70
60
50
40
30
20
10
0
−10

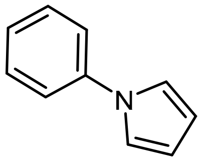

^{1}H NMR

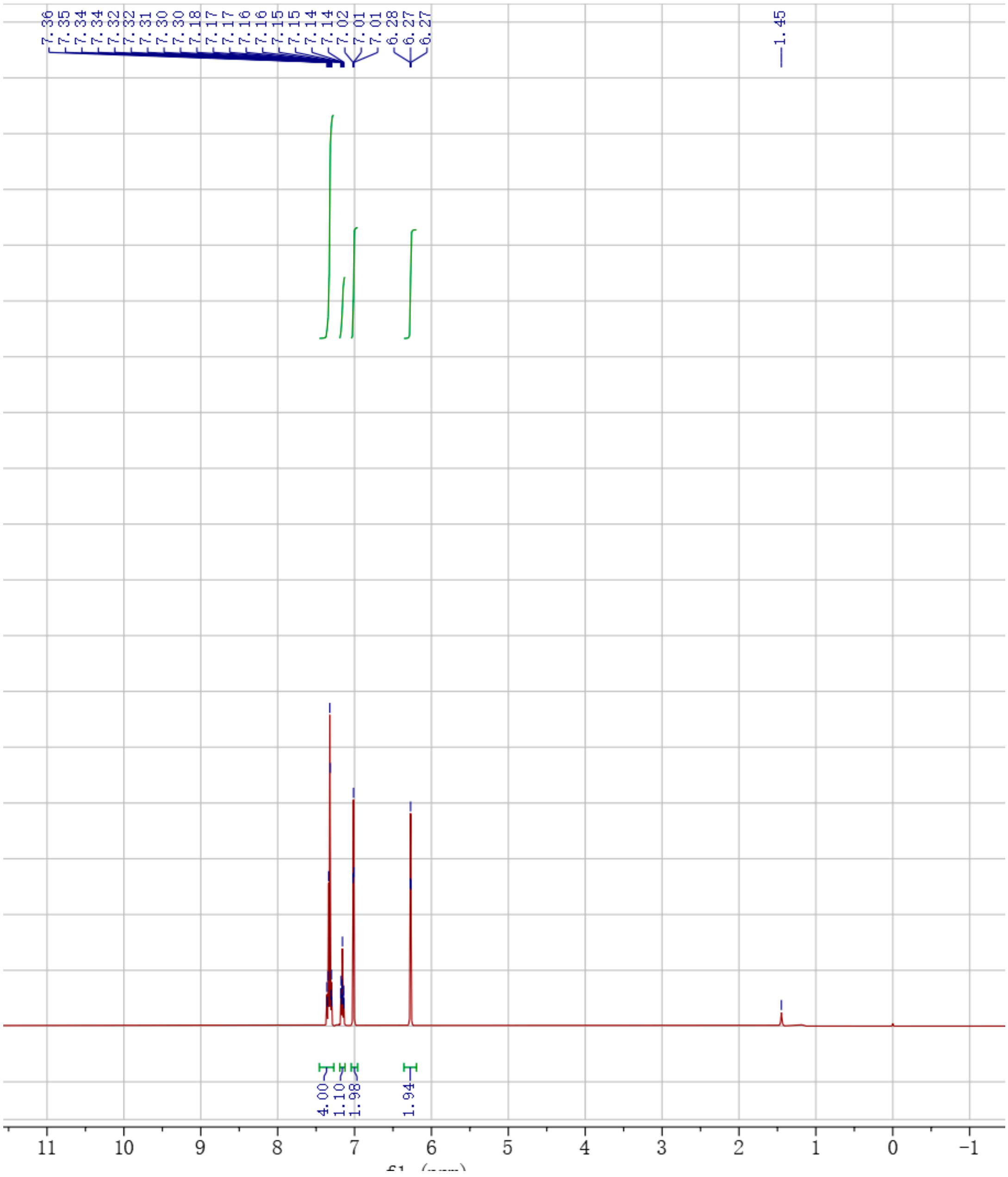

7.36
7.35
7.34
7.34
7.32
7.32
7.31
7.30
7.30
7.18
7.17
7.17
7.16
7.15
7.15
7.14
7.14
7.02
7.01
7.01
6.28
6.27
6.27
1.45
4.00
1.10
1.98
1.94
11
10
9
8
7
6
5
4
3
2
1
0
−1

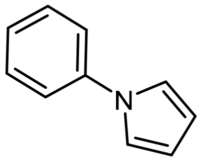

^{13}C NMR

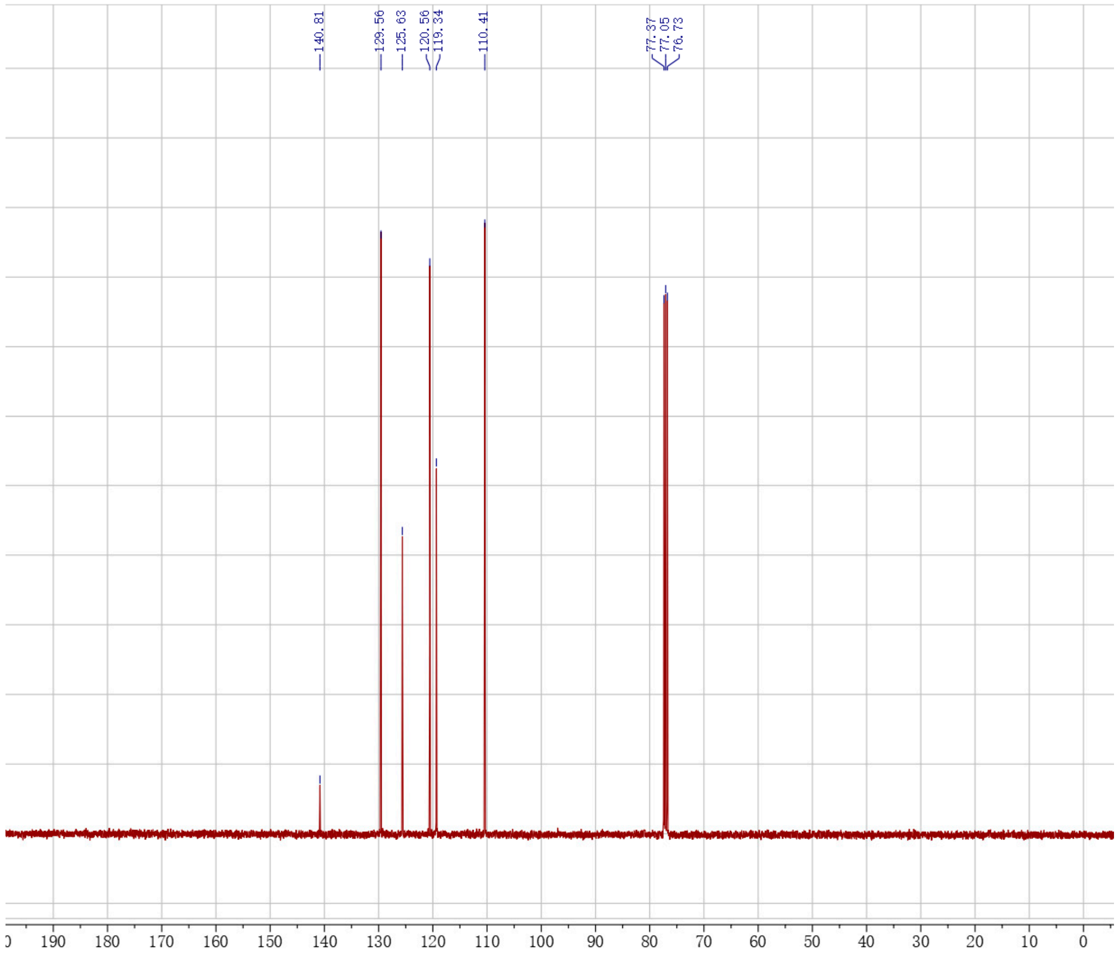

140.81
129.56
125.63
120.56
119.34
110.41
77.37
77.05
76.73
190 180 170 160 150 140 130 120 110 100 90 80 70 60 50 40 30 20 10 0
